# SARS-CoV-2 Infection in Children: Revisiting Host–Virus Interactions Through Post-Infection Immune Profiling

**DOI:** 10.3390/pathogens14090838

**Published:** 2025-08-22

**Authors:** Catarina Gregório Martins, Miguel Ângelo-Dias, Maria de Jesus Chasqueira, Maria João Brito, Tiago Milheiro Silva, Maria Vitória Matos, Maria Teresa Lopes, Hélio Crespo, Mariana Mata, Luís Miguel Borrego, Paulo Paixão

**Affiliations:** 1 Immunology, NOVA Medical School, Faculdade de Ciências Médicas, NMS, FCM, Universidade NOVA de Lisboa, Campo dos Mártires da Pátria, 1169-056 Lisboa, Portugal; 2 CHRC, NOVA Medical School, Faculdade de Ciências Médicas, NMS, FCM, Universidade NOVA de Lisboa, Campo dos Mártires da Pátria, 1169-056 Lisboa, Portugal; 3 Infection, NOVA Medical School, Faculdade de Ciências Médicas, NMS, FCM, Universidade NOVA de Lisboa, Campo dos Mártires da Pátria, 1169-056 Lisboa, Portugal; 4 Unidade de Infeciologia Pediátrica, Hospital D. Estefânia, Unidade Local de Saúde de São José, 1169-045 Lisboa, Portugal; 5 Patologia Clínica, Hospital D. Estefânia, Unidade Local de Saúde de São José, 1169-045 Lisboa, Portugal; 6 Serviço de Imunoalergologia, Hospital da Luz Lisboa, 1500-650 Lisboa, Portugal; 7 Serviço de Patologia Clínica, Hospital da Luz Lisboa, 1500-650 Lisboa, Portugal

**Keywords:** COVID-19, children, immune profile, T-cells, B-cells, specific responses

## Abstract

Children with COVID-19 typically experience milder symptoms and lower hospitalization rates, though severe cases do occur. Understanding age-related immune responses is crucial for future preparedness. We characterized immune response dynamics to SARS-CoV-2 in 145 samples from 119 pediatric patients (<18 years) with confirmed infection, assessed at four distinct time points: <14 days, 14 days–3 months, 3–6 months, and 6–12 months post-infection. At infection, patients presented increased activated T-cells, higher levels of exhaustion (i.e., PD-1^+^), lower numbers of unswitched memory B-cells, and increased antibody-secreting cells (ASCs). Both humoral and cellular anti-SARS-CoV-2 responses increased over time (all patients showed measurable responses in the last assessment). Asymptomatic/mildly symptomatic patients (58.6%) showed increased specific cellular responses from infection onwards, along with enriched memory B-cell subsets (but not ASCs), and distinct T-cell activation profiles. Children with severe disease were younger, predominantly boys, displayed altered T/B-cell ratios, and reduced PHA responses when infected. Compared to adolescents, younger children showed lower antibody titers and weaker cellular responses to SARS-CoV-2, possibly underlining the higher prevalence of severe manifestations in younger children. Our study illustrates important age-, gender-, and disease severity-dependent variations in immune responses to SARS-CoV-2, which can be helpful in improving patient management and immunization strategies adjusted to age groups.

## 1. Introduction

The impact of the recent coronavirus disease 2019 (COVID-19) pandemic on children has been widely discussed. According to current knowledge, it is well established that children with COVID-19 typically present milder symptoms and face a lower risk of hospitalization and life-threatening complications [1,2]. Nevertheless, severe cases do occur, and children can also experience long-term effects (long COVID) or even develop a wide range of post-infection inflammatory conditions, including the Multisystem Inflammatory Syndrome in children, known as MIS-C [2,3,4,5,6,7]. According to the most recent UNICEF report (December 2023), over 17,400 deaths were reported among children and adolescents, representing approximately 0.4% of the global COVID-19 mortality burden. Of these, 47% occurred in children under 9 years of age, and 53% in adolescents aged 10–19 years. As of the end of June 2025, the cumulative global death toll has reached approximately 7.1 million, according to the WHO, but the monthly mortality statistics continue to reflect a consistently low proportion of deaths among children and adolescents. Depending on the study design, the proportions of asymptomatic children range from 16 to 35% [8,9,10,11]. However, these figures are likely underestimated, as asymptomatic children are less frequently tested than those with symptoms, potentially leading to an underestimation of the true prevalence of severe acute respiratory syndrome coronavirus 2 (SARS-CoV-2) infection in pediatric populations.

Several possible explanations emerged as to why COVID-19 in children is less frequent and severe compared to older populations. First, the comorbidities typically associated with severe disease, such as hypertension, diabetes, and chronic lung disease, are less prevalent in young patients. Additionally, children seem to express lower levels of the ACE-2 receptor in nasal epithelia, which can protect them from SARS-CoV-2 initial infection [2,12,13]. Furthermore, possible cross-reactivity or immune training with other coronavirus infections may be frequent in pediatric age. This could be another important factor, since children may present higher levels of such cross-reactive antibodies [12,14]. Protective innate immune factors, such as stronger interferon (IFN) responses, but also higher thymic outputs, allied to T-cell compartments with more regulatory T-cells and lower T-cell exhaustion, could represent additional features that favor children. Somehow, all the factors mentioned above can contribute to the accepted paradigm of milder infectious conditions observed in children; however, this remains a topic of debate [2,15].

In addition, some studies suggest that children were not major vectors in the COVID-19 pandemic, although it is unclear why documented SARS-CoV-2 transmission from children to other children or adults is less frequent [16]. In fact, nasopharyngeal SARS-CoV-2 viral loads in children are comparable to those in other age groups, suggesting they could be as infectious as adults [17]. Other authors, though, claim that children under 10 years old transmit the virus to adults less frequently, whereas older children (10–19 years old) spread the virus to adults at rates similar to adults [18]. Even accounting for viral evolution—from the ancestral strain to the ongoing circulation of various SARS-CoV-2 variants, including those of concern—the aforementioned immune characteristics may also be relevant for reducing transmission rates.

Finally, the duration of immune protection against reinfection with SARS-CoV-2 needs further clarification, in both natural infection and vaccination settings, especially in the pediatric population and in the context of immune-evading variants of concern [19].

Deciphering the immunological profiles of SARS-CoV-2-infected children is critical to understanding the varying degrees of clinical manifestations and age-related mechanisms of susceptibility to SARS-CoV-2. Therefore, in this study, we aimed to characterize circulating immune populations and SARS-CoV-2-specific cellular and humoral immune responses in pediatric patients (<18 years old) to better comprehend the dynamics of the immune function over time, i.e., from acute disease to recovery. Furthermore, by comparing patients according to different age groups and levels of clinical severity, our goal was to identify distinct immunological features, which may be used to further improve patient management and the prompt identification of SARS-CoV-2-associated complications.

## 2. Materials and Methods

### 2.1. Study Design

This observational study enrolled a convenience sample of 119 pediatric patients (aged ≤18 years), from 131 initially enrolled, admitted to Hospital D. Estefânia, Centro Hospitalar Universitário de Lisboa Central (CHULC)/Unidade Local de Saúde de São José, for any indication between April 2021 and May 2022 (Supplementary Figure S1). Inclusion criteria required documented evidence of SARS-CoV-2 infection, either current or prior (within the preceding 12 months), confirmed by laboratory testing (e.g., PCR, antigen testing, and/or IgG seroconversion). Participants were included regardless of the reason for hospitalization, which encompassed a broad spectrum of clinical scenarios, including but not limited to COVID-19-related illness, elective or emergency surgical interventions, and other medical conditions for which SARS-CoV-2 testing was performed as part of institutional admission protocols (Supplementary Table S1). As far as could be determined by the investigators at the time of recruitment, all patients experienced a single episode of SARS-CoV-2 infection. Concomitant primary immunodeficiency was considered an exclusion criterion. Disease severity was categorized according to the recommendations of the Portuguese Directorate General for Health (recommendation 004/2020, updated in recommendation 013/2022) [20], as presented in Table 1. Relevant clinical parameters were recorded for all participants.

According to the Portuguese National Institute of Health Doutor Ricardo Jorge, the relevant SARS-CoV-2 variants circulating in Portugal during this period were Omicron (BA.1, BA.2, BA.3, BA.4, BA.5), Delta (AY.1, AY.4.2, AY.43.5), and Alpha [21].

Patients were further grouped based on age (<10 years vs. ≥10 years old), disease severity (asymptomatic, mild, moderate, and severe disease), and time past infection (i.e., infection < 14 days, T1; 14 d to 3 months after infection, T2; 3 to 6 months after infection, T3; 6 to 12 months after infection, T4).

Asymptomatic and/or mildly symptomatic patients in T1 were recruited from children tested for COVID-19 when admitted to the hospital for conditions with minimal impact on the immune system (i.e., bone fractures). Patients in groups T2, T3, and T4 had the time of infection retrieved from the respective files and hospital records. Longitudinal follow-up assessments in T4 were performed in 10 patients initially recruited at T1.

Written informed consent was obtained from the legal guardians of all participants. The study was approved by the Ethics Committee of Centro Hospitalar Universitário de Lisboa Central/Unidade Local de Saúde de São José (Process No. 1001/2021) and conducted in accordance with the principles of the Declaration of Helsinki.

### 2.2. Immune Assessment

Peripheral blood samples were collected via venipuncture into EDTA-coated tubes, heparinized tubes, and tubes without anti-coagulant from all participants at recruitment. Serum samples were used to assess the humoral immunity (SARS-CoV-2 specific IgGs) using a chemiluminescent immunoassay (Liaison^®^ SARS-CoV-2 TrimericS IgG, DiaSorin Inc., Stillwater, MN, USA) validated for routine use at the Clinical Pathology Laboratory of CHULC.

A comprehensive assessment of cellular immunity was performed by flow cytometry at the Immunology Laboratory of NOVA Medical School. This included basic immunophenotyping procedures to characterize relevant T and B-cell subsets, using the DryFlowEx Activated T-cell (ACT T) Screen Kit and the DryFlowEx Antibody Secreting Cells (ASC) Screen Kit, respectively, both from EXBIO (EXBIO Praha, Vestec, Czech Republic). All protocols followed the manufacturer’s instructions described in the respective kit inserts. The complete list of monoclonal antibodies is provided in Supplementary Table S2.

For T-cells, in addition to the characterization of major subsets, CD4 and CD8 follicular T-cells were assessed, along with different activation markers. B-cell subsets were further classified as transitional, naïve, and memory (switched and unswitched) subsets, along with the characterization of plasmablasts/ASCs.

### 2.3. Assessment of Specific Immune Responses to SARS-CoV-2 Antigens

To evaluate antigen-specific immune responses to SARS-CoV-2, functional assays were used, covering proliferative responses upon stimulation. Proliferative responses were measured using the Act-T4 Cell™ kit (Cytognos, Salamanca, Spain—a BD Biosciences company). This assay involved the stimulation of heparinized peripheral blood cells with phytohemagglutinin (PHA) at 5 µg/mL (Sigma-Aldrich, Budapest, Hungary), and with specific SARS-CoV-2 antigen peptide pools targeting the spike (S) and nucleocapsid (N) proteins (PepTivator^®^ SARS-CoV-2 S and N proteins, MACS Miltenyi Biotec, Bergisch Gladbach, Germany) at a final concentration of 1 µg/mL. According to the manufacturer’s instructions, the incubation period lasted 46–48 h at 37 °C in a 5% CO_2_-enriched atmosphere.

Virus-specific activated CD4 T-cells with proliferative potential (CD3^+^CD4^+^CD25^+^ OX40/CD134^+^) were quantified via flow cytometry after red blood cell lysis. Unstimulated cells were incubated in parallel to serve as negative controls, allowing the calculation of stimulation indexes (SI = [% of CD25^+^CD134^+^ CD4 T-cells in stimulated condition]/[% of CD25^+^CD134^+^ CD4 T-cells in unstimulated condition]).

### 2.4. Flow Cytometry Data Acquisition and Analysis

**Figure 1:**
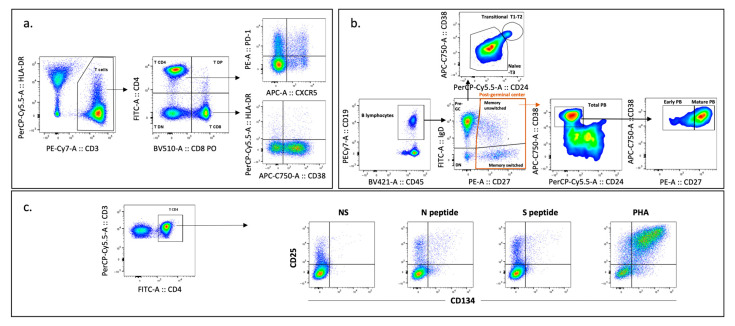
Gating strategy for the flow cytometry studies performed in the study population: (**a**,**b**) Analysis of the circulating T and B-cells compartments. Lymphocytes were identified based on SSC, FSC, and CD45 parameters using Infinicyt^TM^ software, followed by the exclusion of doublets according to FSC-A and FSC-H parameters. Subsequent analyses were performed in FlowJo^TM^ software. T-cells were gated as CD3^+^ and subdivided into CD4^+^ and CD8^+^ populations. Within these subpopulations, CXCR5^+^ PD-1^+^ cells were identified to define follicular CD4^+^ T helper cells (Tfh) and follicular CD8^+^ T-cells (Tfc). Activation status was further assessed through the expression of CD38 and HLA DR. For B-cells, after gating on lymphocytes, CD19+ cells were identified and further characterized into transitional, naïve, memory (switched and unswitched), and plasmablast subsets. (**c**) Analysis of antigen-specific CD4^+^ T-cell responses after stimulation. Expression of CD25 and CD134/OX40 was assessed in CD4^+^ T-cells following stimulation with SARS-CoV-2 S and N Proteins, PHA, and a non-stimulated (NS) condition, to evaluate responses and calculate stimulation indexes. Flow cytometry density plots (FlowJo^TM^) are shown, where colors indicate event density: warmer colors represent higher cell density and cooler colors represent lower density. All images correspond to responses obtained after a 46–48 h incubation period. GC—germinal center.

All acquisitions were performed on a BD FACS Canto II (eight-color configuration), using the FACS Diva software (version 8.0.2). Data analysis was performed with Infinicyt^TM^ (version 2.0) and FlowJo^TM^ (version 10.6.2) software, all from BD Biosciences, San Jose, CA, USA. The gating strategies used for analysis are illustrated in Figure 1.

### 2.5. Statistics

Statistical analyses were performed with GraphPad Prism version 10.0.3 for Windows (GraphPad Software, Boston, MA, USA, www.graphpad.com). Categorical variables were compared using Fisher’s exact test or the Chi-Square test, as appropriate. Comparisons between two independent groups were conducted using either the Student’s t-test or the non-parametric Mann–Whitney U-test. For paired analysis over time in the subgroup of children assessed at both T1 and T4, the Wilcoxon signed-rank test was applied. For comparisons involving three or more independent groups, an analysis of variance (ANOVA) or the Kruskal–Wallis test was used, depending on data distribution, followed by Holm–Šídák’s or Dunn’s multiple comparisons test, respectively. Correlation analyses were performed using the non-parametric Spearman correlation coefficient. A *p*-value < 0.05 was considered statistically significant in all analyses.

To estimate the effect size, we calculated repeated-measures Cohen’s d (*d_RM_*), following Morris and DeShon [22], and using the free online calculators developed by Lenhard and Lenhard [23], available at https://www.psychometrica.de/effect_size.html (accessed on 24 July 2025).

## 3. Results

### 3.1. Characterization of the Study Population

To decipher modifications in the immunological profiles of SARS-CoV-2-infected children, our study recruited 74 children and adolescents with confirmed SARS-CoV-2 infection within the previous 14 days (T1), and 71 with documented infection occurring more than 14 days prior (23 recruited 14 d-3 months after infection, T2; 20 recruited 3–6 months after infection, T3; and 28 recruited 6–12 months after infection, T4).

Overall, this population had a median age of 6.5 years (ranging from 6 days to 17 years), with 40% being girls. Most patients were asymptomatic (30.6%) and/or presented mild symptoms (28.5%). Additionally, 7.6% of patients showed moderate symptoms, and 33.3% presented severe disease manifestations. Demographic and clinical characteristics of each group are detailed in Table 2. Overall, there were no significant variations in age and disease symptoms between groups, although more boys were recruited at the third time point (T3).

### 3.2. Immune Profile After SARS-CoV-2 Infection in Pediatric Patients

**Figure 2:**
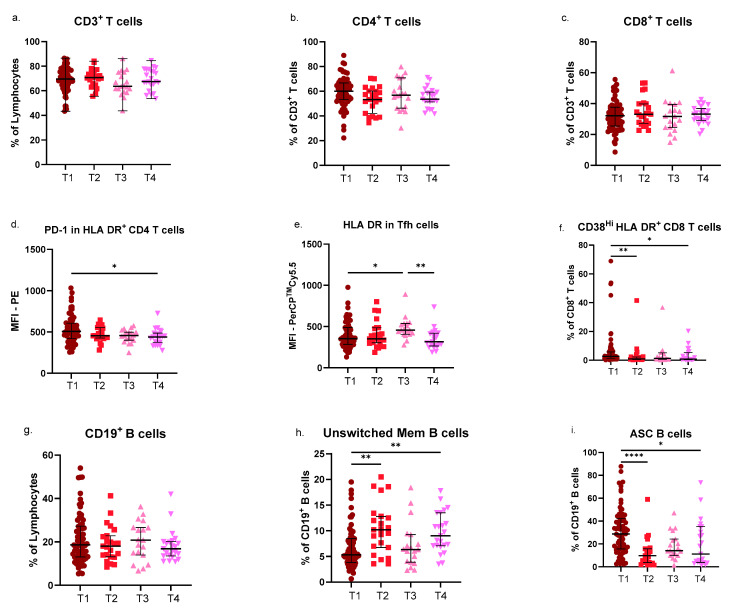
Variations in lymphocyte subsets at different timepoints after SARS-CoV-2 infection: (**a**–**f**) Scatter dot plots showing individual points with median and interquartile range for T-cells, CD4 T-cells, CD8 T-cells, PD-1 expression in activated CD38^−^ HLA DR^+^ CD4 T-cells, HLA DR expression in CXCR5^+^ PD-1^+^ Tfh cells and activated CD38^Hi^ HLA DR^+^ CD8 T-cells in the four groups of patients. (**g**–**i**) Scatter dot plots with individual points and median and interquartile range for B-cells, unswitched memory B-cells, and ASC in the four groups of patients. Group comparisons were performed with the non-parametric Kruskal–Wallis test, followed by Dunn’s multiple comparisons test. ASC, antibody secreting cells; MFI, mean fluorescence intensity; Unswitched Mem, unswitched memory; T1, time point 1; T2, time point 2; T3, time point 3; T4, time point 4; * *p* < 0.05; ** *p* < 0.01; **** *p* < 0.0001.

The initial approach was set to explore the impact of SARS-CoV-2 infection in several immune subsets, including CD4 T-cells, CD8 T-cells, and B-cells. We found no major differences in the circulating subsets of CD4 T-cells in children assessed at time points T1, T2, T3, and T4. However, the expression levels of PD-1 in HLA DR^+^ (activated) CD4 T-cells were higher in infected patients (*p* = 0.0183, Kruskal–Wallis), which gradually decreased over time, achieving statistical significance only at T4 (*p* = 0.0276) (Figure 2d). Thus, during infection, activated CD4 T-cells show signs of exhaustion, which are reduced upon resolution. Additionally, HLA DR expression in Tfh differed significantly across time points (Figure 2e). In fact, the levels of this activation marker in Tfh cells increased up to T3 (*p* = 0.0330) and then decreased in T4 (*p* = 0.0062). Again, infection seems to drive activation pathways of specific subsets, such as follicular T-cells. This effect may be prolonged in time and eventually affect other responses, as we know Tfh cells modulate B-cells and germinal center reactions. Full data are available in Supplementary Table S3.

Although no significant differences were observed in PD-1 or HLA DR expression levels within CD8^+^ T-cells, unlike their CD4^+^ counterparts, the proportion of activated CD8^+^ T-cells varied over time. Specifically, children exhibited higher levels of CD38^−^ HLA DR^+^ CD8^+^ T-cells (*p* = 0.0335) and CD38^Hi^ HLA DR^+^ CD8^+^ T-cells (*p* = 0.0009) during acute infection (Figure 2f), suggesting that the virus-driven activation also affects cytotoxic T-cells, as expected, eventually without interfering with immune exhaustion mechanisms in these cells.

In line with this, the circulating B-cell compartment also showed notable changes, particularly in more differentiated subsets, known to respond after antigenic stimulation. First, unswitched memory B-cells were significantly decreased during infection, compared to both T2 (*p* = 0.0028) and T4 (*p* = 0.0047). Their levels at T3 were similar to those observed at T1. On the other hand, circulating ASC were significantly increased during infection, particularly when compared to T2 (*p* < 0.0001) and T4 (*p* = 0.0311), probably as a result of direct and indirect activation mechanisms driven by the virus.

### 3.3. Specific Humoral and Cellular Responses to SARS-CoV-2 in Children After Infection

After identifying activation events in the immunophenotypic assessment, our goal was to further assess the dynamics of specific responses against the SARS-CoV-2 S and N proteins, performed in parallel with non-specific responses to PHA. Children with ongoing infection presented lower percentages of CD4 T-cells with proliferative potential (CD25^+^ CD134/OX40^+^) after PHA stimulation (*p* = 0.0024; Supplementary Table S3). These levels increased over time, showing significant recovery at T3 (*p* = 0.0295) and T4 (*p* = 0.0221). Similarly, stimulation indexes followed this trend, with a decrease in T1 compared to later time points (*p* = 0.0584 vs. T3; *p* = 0.0175 vs. T4).

In contrast, the proliferative responses to SARS-CoV-2 S and N proteins did not show significant variations in the percentages of cells with proliferative potential. However, stimulation indexes for the N protein showed a modest but significant increase following infection. Specifically, lower responses were seen at T1 compared to subsequent time points (*p* = 0.0311), while stimulation indexes for the S protein remained stable throughout. Nonetheless, the distribution of responses to protein S at T1 segregated lower and higher responders, and we observed positive correlations with age for both protein S and protein N responses in the initial time point (S, r = 0.6347, *p* < 0.0001; N, r = 0.3760, *p* = 0.0017; Spearman correlation), but not with time post-infection at T1, which shows that younger children may have lower specific responses at start.

**Figure 3:**
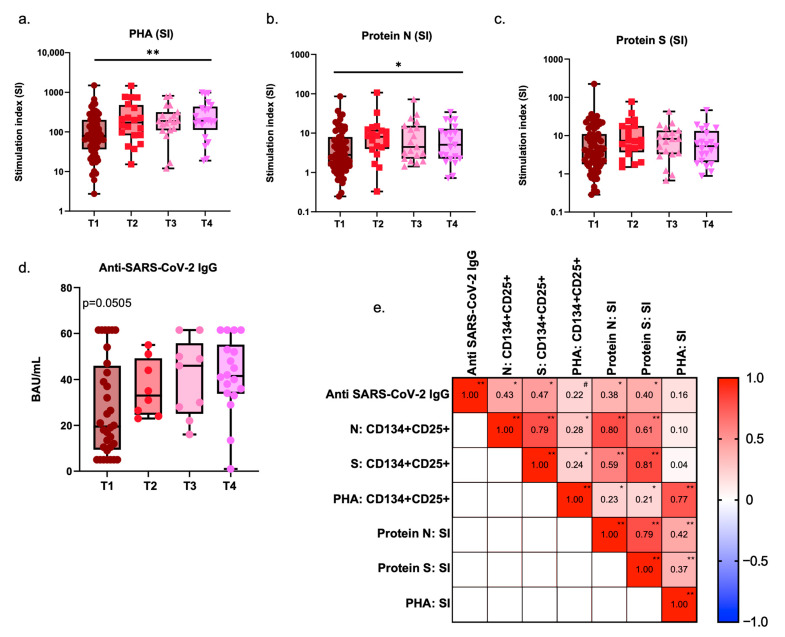
Specific SARS-CoV-2 cellular and humoral responses in children: dynamics post-infection and correlation matrix: (**a**–**c**) Box-and-whiskers plots with individual points and median and interquartile range for Stimulation Indexes of CD25^+^ CD134^+^ CD4 T-cells activated after a 46–48 h incubation period with PHA, SARS-CoV-2 S and N proteins. Group comparisons were performed with the non-parametric Kruskal–Wallis test; * *p* < 0.05; ** *p* < 0.01. (**d**) Box-and-whiskers plot with individual points and representations of median and interquartile range for SARS-CoV-2-specific IgG antibodies. Group comparisons were performed with the non-parametric Kruskal–Wallis test. (**e**) Correlation matrix with Spearman’s r correlation indexes for Anti SARS-CoV-2 IgG (BAU/mL), specific cellular responses measured for SARS-CoV-2 N and S proteins (% and stimulation indexes), and for PHA responses, considering the values for all patients and at all time points. In this matrix, * is used to identify *p* < 0.05; ** for *p* < 0.0001, and # for *p* < 0.1. PHA, phytohemagglutinin; SI, stimulation index; T1, time point 1; T2, time point 2; T3, time point 4; T4, time point 4.

The levels of specific IgG antibodies against SARS-CoV-2 behaved similarly to the N protein, showing a trend towards increasing titers over time (*p* = 0.0505). Furthermore, when considering all patients, regardless of post-infection timing, specific IgG titers were positively correlated with the percentages of CD25^+^ CD134^+^ CD4 T-cells responding to N (r = 0.4285, *p* < 0.0001) and S proteins (r = 0.4706, *p* < 0.0001), but also with their respective stimulation indexes (N: r = 0.3755, *p* = 0.0024; S: r = 0.4007, *p* = 0.0011). Additionally, strong positive correlations were observed between N and S responses, either looking at each group separately or the whole population, which is somehow expected as both are presented to the immune system in the context of an infection (Figure 3).

### 3.4. Longitudinal Analysis of Immune Dynamics Following SARS-CoV-2 Infection

Recognizing the importance of longitudinal analyses with the same patients, we further explored a subgroup of ten patients who had been assessed for infection at T1 and at T4 (i.e., at least 6 months after infection). This group included six girls and four boys, with a mean age of 12.10 (8.31) years, of which three were asymptomatic, three had mild symptoms, one had moderate symptoms, and three had severe manifestations.

**Figure 4:**
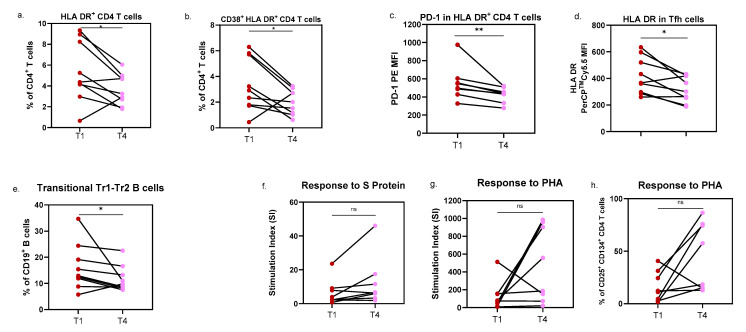
Variations in lymphocyte subsets in patients followed up after 6 months of SARS-CoV-2 infection. (**a**–**e**) Estimation plots with individual points for each patient monitored at infection (T1), and after 6 months of infection (T4), showing the variations of activated HLA DR^+^ and CD38^+^ HLA DR^+^ CD4 T-cells, PD-1 expression in activated CD38^−^ HLA DR^+^ CD4 T-cells, and HLA DR expression in Tfh cells. (**e**) and transitional Tr1-Tr2 B-cells. (**f**–**h**) Estimation plots displaying individual changes in stimulation indexes for SARS-CoV-2 S protein and PHA, as well as the frequency of proliferating CD4 T-cells (CD25^+^ CD134^+^) after PHA stimulation, between T1 and T4. Group comparisons were performed with the Wilcoxon signed-rank test. MFI, mean fluorescence intensity; PHA, phytohemagglutinin; SI, stimulation index; * *p* < 0.05; ** *p* < 0.01; ns, non-significant.

We were also able to confirm the observations made for HLA DR expression in Tfh cells (*p* = 0.0195; *d_RM_* = −0.897, with a confidence interval of −2.172 to −0.233) and PD-1 expression in activated HLA DR^+^ CD4 T-cells (*p* = 0.0039; *d_RM_* = −1.714; 95% confidence interval [CI] = −3.815 to −1.655), both increased at infection. Additionally, the percentage of CD38^+^ HLA DR^+^ CD4 T-cells (*p* = 0.0494; *d_RM_* = −0.684; 95% CI = −1.769 to −0.133) and total HLA DR^+^ CD4 T-cells (*p* = 0.0404; *d_RM_* = −0.808; 95% CI = −2.105 to −0.183) decreased from T1 to T4, (Figure 4a,b).

On the contrary, only non-significant trends were observed for activated CD8 T-cells, with higher levels of CD38^+^ HLA DR^+^ CD8 T-cells and CD38^Hi^ HLA DR^+^ CD8 T-cells in infected children. Regarding B-cells, differences were limited to the Tr1-Tr2 transitional subset, with increased percentages detected during infection (*p* = 0.0391; *d_RM_* = −0.620; 95% CI = −1.783 to 0.108). In brief, despite the small number of patients included in this longitudinal follow-up, the effect size analyses performed suggest substantial changes from T1 to T4.

PHA-induced proliferative responses were again decreased at T1 (*p* = 0.0547), while the stimulation indexes for S protein tended to increase over time (*p* = 0.0547). Notably, two patients showed slightly lower S protein responses 6 months after infection; both were asymptomatic at T1.

Only three of these patients completed the antibody assessment, preventing meaningful analysis of humoral response in this setting.

### 3.5. Immune Responses and Severity of Disease Symptoms

To uncover immune variations related to disease severity, we further assessed immune responses in patients showing different symptoms. For that, patients recruited at T1 (n = 74) were divided into four major groups: asymptomatic, mild symptoms, moderate to severe symptoms, and those who developed MIS-C. Overall, the asymptomatic group included more girls and had a higher median age compared to other groups (Table 3).

**Figure 5:**
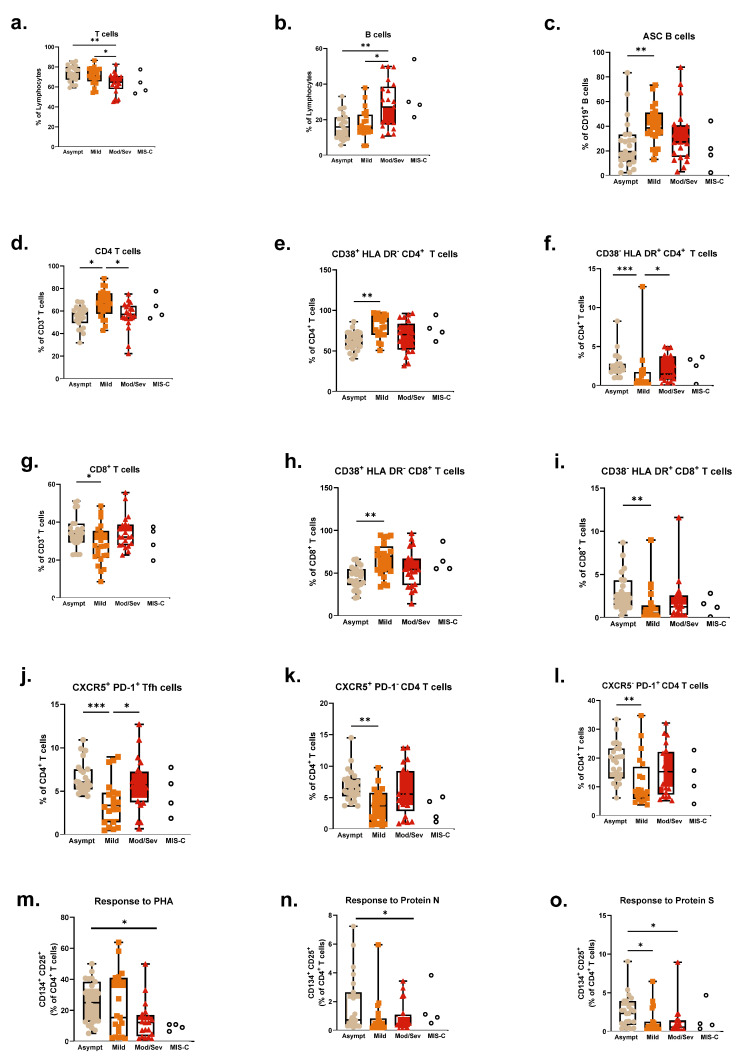
Lymphocyte subsets and specific responses to SARS-CoV-2 according to disease severity at infection. (**a**–**l**) Scatter boxplots with individual points for each patient monitored at infection, according to disease severity (i.e., asymptomatic, mild, moderate/severe, and MIS-C), showing the circulating percentages of T-cells, B-cells, ASC, CD4 T-cells and activated subsets, CD8 T-cells and activated subsets, and CXCR5^+^ PD-1^+^ Tfh cells and related subsets. (**m**–**o**) Scatter boxplots with individual points for each patient monitored at infection, according to disease severity (i.e., asymptomatic, mild, and moderate/severe/MIS-C), showing the proliferative responses upon PHA, SARS-CoV-2 N protein, and S protein stimulation, by activated proliferating CD4 T-cells (CD25^+^ CD134^+^). Group comparisons were performed with the non-parametric Kruskal–Wallis test. ASC, antibody-secreting cells; Asympt (beige), asymptomatic patients; Mild (orange), mild patients; Mod/Sev (red), moderate/severe; MIS-C—Multisystem Inflammatory Syndrome in Children; PHA, phytohemagglutinin; Tfh, follicular T helper cells. * *p* < 0.05; ** *p* < 0.01; *** *p* < 0.001.

As per the immune profile, several differences were observed in T-cells, particularly within the CD4 compartment when dividing patients according to disease severity (Figure 5a,d–f). Severe patients presented lower levels of total T-cells (*p* = 0.0015), while CD4 T-cells were decreased in both severe and asymptomatic groups, compared to patients with mild symptoms (*p* = 0.0028). Similarly, levels of activated CD38^+^ HLA DR^−^ CD4 T-cells and overall CD38 expression were also decreased in both severe (*p* = 0.0008) and asymptomatic (*p* = 0.0019) patients, while CD38^−^ HLA DR^+^ CD4 T-cells were increased in these groups compared to the mild symptoms group (*p* = 0.0006).

Tfh cells (CXCR5^+^ PD-1^+^), as well as CXCR5^+^ PD-1^−^ CD4 T-cells (*p* = 0.0041) and CXCR5^−^ PD-1^+^ CD4 T-cells (*p* = 0.0061), were significantly increased in asymptomatic patients (*p* = 0.0005), particularly compared to mild patients (Figure 5j–l). Recalling the impact of follicular subsets in other immune functions like antibody secretion, this increased presence may be related to superior responses in the asymptomatic group.

Mild patients also showed distinct patterns in the CD8 compartment, with lower percentages of CD8 T-cells (*p* = 0.0137) compared to asymptomatic and severe patients. However, the asymptomatic group presented lower levels of activated CD38^+^ HLA DR^−^ cells, along with higher levels of activated CD38^−^ HLA DR^+^ CD8 T-cells (*p* = 0.0021 and *p* = 0.0014, respectively), as per Figure 5g–l, in what seems to be a conjugation of different response mechanisms in patients with different levels of severity.

Severe patients presented increased percentages of circulating B-cells (*p* = 0.0044). Asymptomatic patients, though, had fewer pre-germinal center/naïve B-cells but showed increased unswitched and switched memory subsets compared to both mild and moderate–severe patients (*p* = 0.0001, *p* = 0.0002, and *p* = 0.0005, respectively), in line with the TFH observations. Within pre-germinal subsets, mild patients had particularly augmented Tr1-Tr2 transitional cells (*p* = 0.0038) with lower Tr3 cells than the other groups (*p* = 0.0038). Finally, ASCs were also lower in the asymptomatic patients (*p* = 0.0056), despite no differences in specific antibody titers being found between groups.

The assessment of specific T-cell responses revealed higher levels with both S and N proteins (*p* = 0.0024 and *p* = 0.0393, respectively) in asymptomatic patients. The differences observed for S protein were particularly notable, with significant differences between both mild and moderate–severe, while N responses were augmented in asymptomatic patients only when compared to mild patients. Unspecific responses to PHA were decreased in moderate–severe patients, particularly when compared to the asymptomatic group (*p* = 0.0352), which may underline a compromised response in this group. For illustrative purposes, patients who were recruited during infection (T1) and further developed MIS-C (n = 4) are also presented in the graphs.

### 3.6. Impact of Age and Gender on the Immune Response to SARS-CoV-2 Infection

Considering the important modifications observed in the immune system during the first years of life and the wide range of ages included in our study, we further assessed how age could affect the immune patterns observed in the context of SARS-CoV-2 infection. To this end, we stratified patients into two groups: children (<10 years old, n = 44) and adolescents (≥10 years old, n = 33). A greater proportion of children developed severe symptoms or MIS-C (32.6%) compared to adolescents (15.2%, *p* = 0.0091). Furthermore, children showed lower percentages of T-cells than adolescents (*p* = 0.0063), with more CD4 and less CD8 T-cells (*p* = 0.0084 and *p* = 0.0131, respectively), increased activated CD38^+^ HLA DR^−^ (*p* < 0.0001) and HLA DR^+^ CD4 T-cells (*p* = 0.0052) and increased expression levels of CD38 (*p* = 0.0002). PD-1 expression, though, was higher in adolescents (*p* = 0.0172), which also showed increased percentages of PD-1^+^ cell subsets, i.e., CD4 CXCR5^−^ PD-1^+^ (*p* < 0.0001). Tfh subsets, CXCR5^+^ PD-1^+^ and CXCR5^+^ PD-1^−^, were also decreased in children (*p* = 0.0042 and *p* < 0.0001, respectively).

Among CD8^+^ T-cells, only the proportions of activated subsets differed significantly between groups: children exhibited higher percentages of CD38^+^ HLA-DR^−^ CD8^+^ T-cells, whereas adolescents showed higher percentages of CD38^−^ HLA-DR^+^ CD8^+^ T-cells (*p* < 0.0001 for both comparisons). Like their Tfh counterparts, CXCR5^+^ PD-1^+^ Tfc were decreased in children (*p* = 0.0057). In brief, children have decreased follicular T-cells and lower T-cell exhaustion.

Opposite to total T-cells, total B-cells (*p* = 0.0004), as well as naïve B-cells (*p* = 0.0062), were more abundant in children than adolescents, though the germinal center compartment was mostly composed of Tr3 transitional B-cells in both subgroups. Still, children presented higher percentages of transitional Tr1-Tr2 and lower transitional Tr3 B-cells than adolescents (*p* = 0.0004). In line with this, memory B-cells were also decreased in children (*p* = 0.0048), which similarly presented decreased percentages of mature ASC and switched memory B-cells (*p* = 0.0066 and *p* = 0.0027, respectively). As expected, percentages of early ASCs were increased in children compared to adolescents (*p* = 0.0037), while CD21^+^ double-negative B-cells were diminished in children (*p* = 0.0001). Following the trends of switched memory and mature ASCs subsets, lower titers of SARS-CoV-2-specific antibodies were observed in children compared to adolescents (*p* < 0.0001).

**Figure 6:**
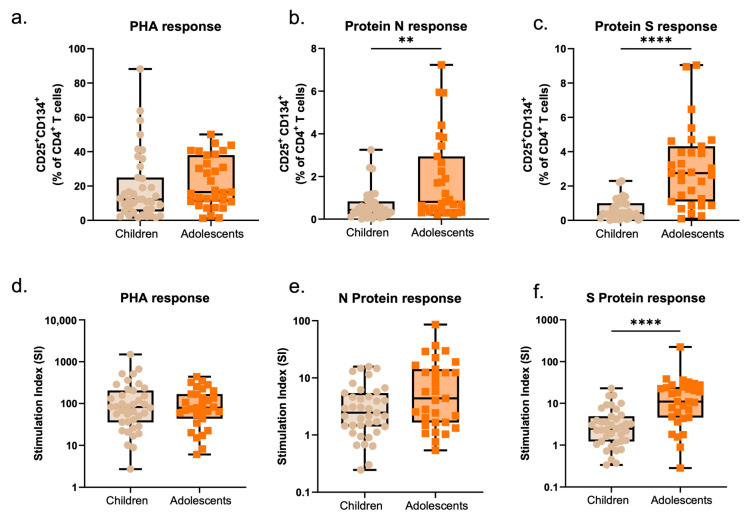
Specific SARS-CoV-2 cellular responses in children and adolescents: (**a**–**f**) Estimation boxplots with individual points for children and adolescents assessed at infection (T1), showing the variations in activated proliferating CD4 T-cells (CD25+ CD134+) upon PHA stimulation, and stimulation with SARS-CoV-2 N and S proteins. Comparisons between groups were conducted with the non-parametric Mann–Whitney U-test. PHA, phytohemagglutinin. ** *p* < 0.01; **** *p* < 0.0001.

As per cellular-specific responses, once again, children showed lower intensities compared to those observed in adolescents, considering responses to S (*p* < 0.0001, for percentages of CD25^+^ CD134^+^ CD4 T-cells and stimulation indexes) and N proteins (*p* = 0.0016, for percentages of CD25^+^ CD134^+^ CD4 T-cells and *p* = 0.0521 for stimulation indexes). No differences were reported for unspecific PHA stimulation between children and adolescents (Figure 6). Still, either considering all time points or time point 1 separately, strong correlations were observed between specific responses to SARS-CoV-2 proteins and age. Compared to younger children, adolescents show, through time (from T1 to T4), higher levels of response, considering both specific T-cell responses and antibody levels (Supplementary Figure S2).

We also assessed the effects of gender on the immune responses of our patients. Globally, there were no differences in age or COVID-19 symptoms between boys (n = 41) and girls (n = 36), neither in the T-cell compartment, nor in specific responses. However, we encountered differences in the B-cell compartment. Indeed, at infection (T1), girls showed increased values of unswitched memory B-cells (*p* = 0.0058) and decreased post-germinal ASC (*p* = 0.0266) compared to boys.

## 4. Discussion

The SARS-CoV-2 pandemic has posed unprecedented challenges to the global medical and scientific communities. In pediatric populations, the full scope and impact of COVID-19 remain under investigation, particularly in light of the evolving landscape of viral variants and the heterogeneous vaccination strategies implemented across countries.

In line with recent reports, we observed that the classical paradigm defining less severe manifestations of COVID-19 in children was observed in our population, with more than 50% of patients being either asymptomatic or showing mild symptoms. However, compared to other studies [2,5,6], a greater proportion of our patients exhibited severe clinical manifestations. This reflects the referral nature of our center, a pediatric central hospital to which severe patients from all of southern Portugal and the islands are referred.

One of the objectives of our study was to document the evolution in time of the immune profiles in children infected with SARS-CoV-2. Children can assemble robust humoral and cellular immune responses to SARS-CoV-2, with possible heterogeneity in the level of response according to disease severity or age [24,25]. Despite some studies that have approached this same goal, data regarding the evolution in time of specific immune subsets is still scarce. A broader comprehension of these dynamics may be very important to understand the development of memory and specific immune responses, but also to enlighten post-COVID-19 complications in children, like MIS-C, or other gastrointestinal or neurological manifestations [7,25,26]

As reported by other authors, a decrease in the circulating T-cell compartment and in the T/B ratios is paramount to active SARS-CoV-2 infection [25,27,28,29]. Even though we did not compare our patients with age-matched healthy controls nor found differences in T and B-cells over time, our study shows decreased levels of T-cells and increased levels of B-cells in severe patients, compared to those with absent/milder symptoms. Beyond serving as indicators of ongoing infection a marker of ongoing infection, T/B-cell ratios may also represent markers of severe forms of the disease, as has been described for MIS-C patients [27,28].

Another feature of acute infection in our study was the high levels of activation and exhaustion markers, such as HLA DR, CD38, or PD-1. As in any other acute condition, increased levels of immune activation would be expected along with SARS-CoV-2 infection. However, activation profiles differed between CD4 and CD8 T-cells. Moreover, in the CD4 T-cell population, the expression levels of HLA DR kept rising until the sixth month post-infection, even though the percentage of activated cells did not change significantly. As reported by Govender and colleagues [30], other activation markers—like CD69—were more broadly increased, not only during hospitalization. Also, high levels of CD69^+^ CD4 T-cells persisted until the sixth and seventh months after infection in adult patients. This sustained level of activation may contribute to the development of late complications, as reported in both children and adults. In severe COVID-19 patients, overactivated CD8 T-cells have been correlated with systemic inflammation and cytokine storm, along with tissue injury and immune disorders [31].

Other authors addressed T-cells in adults and children infected with SARS-CoV-2, reporting more T-cells in children, as they have increased thymic outputs, but also lower levels of T-cell exhaustion [15,32]. Our data seem to support the observations on cell exhaustion, but further complete them with a finer characterization of different age groups within children. Indeed, we report the increase with age of exhaustion markers like PD-1 (i.e., in adolescents). Still, as time went by, both activation and exhaustion levels diminished significantly. Accordingly, a comprehensive longitudinal analysis of mild/asymptomatic adults and children performed by Khoo and colleagues [32] reported a smaller footprint imposed by the infection in children, and also a post-infection recovery in the levels of activated and exhausted T-cell subsets. The severity of infection can, however, interfere with this normalization, particularly in what concerns exhaustion, as suggested by other studies [33]. Adults with severe COVID-19 show high levels of T-cell exhaustion, sustained in time, and this has been considered a risk factor for reinfection and for the development of other pathologies [33]. Nonetheless, a different scenario may be possible in children. Transcriptome-wide studies implicated the downregulation of exhaustion levels in cytotoxic cells (NK and CD8 T-cells) with a sustained inflammatory environment, enhancing autoreactivity and being involved in the pathogenesis of MIS-C [34].

Compared to other respiratory infections, an exclusive pathological immune signature with signals of T-cell exhaustion, particularly in CD4 T-cell populations, has been suggested in adult patients with severe COVID-19 [35]. Here, we found no differences in PD-1 expression in pediatric patients with different severities of infection, except for higher percentages of CXCR5^−^ PD-1^+^ CD4 T-cells in asymptomatic children. Interestingly, other activation markers exhibited comparable patterns between severe and asymptomatic pediatric cases. Whether these similarities reflect intrinsic immunological features or are influenced by external variables, such as therapeutic interventions or age-related factors in the more severely affected patients, remains to be determined. Furthermore, the low number of patients in the latter subgroups did not allow us to undergo a robust follow-up in time according to disease severity. Thus, comprehensive studies that consider a wide age range of pediatric patients associated with diverse disease severity manifestations are still needed. These would allow us to better comprehend how cell activation and exhaustion are modulated by infection and lead to COVID-19-derived complications, such as MIS-C, or, on the contrary, how the occurrence of more severe manifestations may trigger immune activation and exhaustion.

The dynamics of B-cell subsets post-infection were also interesting in our pediatric cohort. In our patients, infection was associated with increased ASCs, but also with lower levels of circulating unswitched memory B-cells, which increased afterwards and recovered with time. In fact, the important presence of ASCs in acute infectious conditions would be expected, and other authors have already confirmed a rapid increase in clonal expansion of B-cells in response to SARS-CoV-2 infection [36]. Nevertheless, in normal responses to different microorganisms, the period that ASCs stay in circulation can vary. Moreover, the rapidity of appearance may be associated with antibody duration, or even with disease severity, eventually in an age-dependent manner [37].

Indeed, adults infected with SARS-CoV-2 who produce higher amounts of ASC specific for the S protein seem to develop severe forms of the disease, while this association was not observed in children [37,38]. However, our pediatric patients presented high amounts of ASC at infection, particularly switched ASC, with loss of IgD, an observation that could be in line with the recently reported distinctive magnitude of interferon-stimulated genes in B-cells from SARS-CoV-2-infected children [39].

Furthermore, when looking at disease severity, our data are in line with other observations in adults, showing lower levels of ASC in asymptomatic children, thus, corroborating the idea that the quality of the B-cell responses in SARS-CoV-2 infection may affect disease course, in adults and children [40], or be affected by it. Another important topic to address is the fact that different variants were active throughout the duration of our study. The real clinical impact of these variants in young children is still the object of study [41,42]. Nonetheless, studies over the impact on the immune system support the idea that earlier variants (e.g., Alpha, Delta) tend to induce broader and more robust neutralizing antibody responses than Omicron, which is associated with more localized upper respiratory tract infection and relatively milder immune stimulation [43]. Of course, these differences may influence not only durability but also the immune profile over time in our patients.

Gender does not appear to exert a significant influence on the humoral immune response to SARS-CoV-2 infection, as measured by IgG binding activity and neutralizing antibody titers [43]. In line with this, our results did not show important differences between boys and girls infected with SARS-CoV-2 from the immune point of view. Still, subtle modifications in the B-cell compartment may be present at disease onset. In fact, to our knowledge, our study is the first to identify lower levels of ASC cells in infected girls, who are often asymptomatic or show milder disease forms compared to boys, as reported before for children and adults [44]. In children, B-cell responses seem to be relevant in terms of disease outcome and could be among the factors that favor girls in the context of COVID-19.

Nonetheless, looking at the impact of age within pediatric ranges, we observed variations, including in ASC when comparing children below 10 years old to adolescents. In fact, we reported several differences between children and adolescents, regarding both subset characterization (i.e., lower T-cells, higher CD4/CD8 T-cell ratios, increased activation levels in T-cells, along with decreased switched ASC values for this subset in children) and specific anti-SARS-CoV-2 responses (with children showing low antibody titers and lower cellular responses).

Another important point to consider in the context of pediatric patients is that both severe manifestations and MIS-C were more frequent in children than in adolescents, as reported previously by Dong and colleagues [45] and now reinforced by the data from our cohort. Indeed, a recent application of a machine-learning approach in a pediatric cohort identified a higher risk of experiencing a greater number and longer duration of symptoms in younger children, with some possible effects of variants like Omicron [41]. Other studies have investigated the causes of the disease course in children and have addressed the immune profiles, considering both innate and adaptive immune responses [1,15]. Indeed, milder infections could result from a stronger innate immune system, along with a more effective presence of lymphocytes [46]. Children of younger ages seem to be more prone to serious infections, and as mentioned before, looking at their immune profile, we observed lower percentages of circulating T-cells, with increased CD4/CD8 T-cell ratios. Furthermore, the T-cells of younger patients did show increased levels of activated T-cells, though with lesser exhaustion levels, according to the expression of PD-1.

Additionally, follicular T-cells, essential for humoral responses, were also decreased in young children, which could result in, or at least relate to, the alterations observed in B-cells of younger children. For all the above, we believe that, in the future, it would be beneficial to address a more detailed characterization of pediatric patients with finer age divisions, since not only immune differences are observed, but the literature, and this study, document more serious complications and outcomes in younger patients, that may have an underlying immune background.

SARS-CoV-2-specific responses have been under investigation since the beginning of the pandemic, in both natural and artificial immunization settings. So far, studies have shown that it is possible to identify SARS-CoV-2-specific T-cells around 2 days after the beginning of symptoms, possibly with the CD4 T-cell peak preceding their CD8 counterparts [47,48,49]. Moreover, SARS-CoV-2-specific T-cell responses increase with time post-infection and age, with several pieces of evidence that these responses persist over time after a single infection with SARS-CoV-2, maintaining robustness for long periods (>9–12 months) in adults and children [50,51,52,53]. Amongst the different options available, the characterization of CD25 and CD134 co-expression allows the identification of antigen-specific CD4 T-cells, which are responding to the stimulation, thus assessing specific activation and proliferation responses [32,54]. Using similar approaches to ours, Khoo and colleagues observed that responses to S protein increased in adult patients from acute infection to the convalescing period; however, the differences in children were not significant [32]. Indeed, our data are in line with these, showing an increasing trend, though, in responses to N and S proteins in the assessments after infection (i.e., T2, T3, and T4). However, it was interesting to see that upon infection, patients were showing lower levels of response to PHA, a mitogenic agent. Thus, proliferative responses seem to be hampered at infection, and this is even more evident in patients presenting more severe manifestations of the disease, as well as complications like MIS-C.

Other studies revealed that mild forms of COVID-19 show early induction of specific T-cells (within 7 days of symptoms) [55], and that the rapid induction, magnitude, and breadth of responses can be associated with accelerated viral clearance and mild COVID-19, or, on the contrary, with severe forms of the disease [29,56]. Often, these studies are conducted in adult populations and/or include limited proportions of pediatric patients [57]. Thus, it is important to explore the implications of such observations in children and even address differences in distinct ranges of pediatric patients. In fact, in line with other reports [47,49,56], our asymptomatic pediatric patients were the ones presenting higher levels of specific cellular responses at infection, considering both S and N proteins. Moreover, we observed increased responses to the S protein at infection in adolescents, compared to younger children, complementing previous observations by Cohen and colleagues [50]; however, we also report a sustained lower level of both humoral and cellular responses in young children after infection. Briefly, the study of specific responses can be relevant for the assessment of disease outcomes at these ages or even for exploring immunization strategies.

Limitations to our study must be acknowledged as well. First, the use of a convenience sample, largely dictated by restricted access to hospital facilities during the COVID-19 pandemic, limited our ability to recruit a more balanced and systematically matched cohort. The hospital setting during the study period (April 2021 to May 2022) was subject to stringent public health measures, which impacted the feasibility of enrolling a broader or more representative pediatric population, including the inclusion of a healthy control group. Additionally, parental reluctance, particularly regarding repeated blood sampling in young children, posed a significant challenge.

Second, while the data presented here are valuable, we must acknowledge that our study would have benefited from a larger sample size to enrich subgroup evaluation. Certainly, a greater number of participants with a proper longitudinal assessment over time would have allowed us to strengthen our conclusions and even perform more stratified analyses according to age groups or gender. The concordance between results obtained in cross-sectional and paired analyses of our cohort somehow reinforces the validity of our findings. Nonetheless, increased recruitment, particularly in T2, T3, and T4, would have made it possible to further dissect how the severity of clinical symptoms might affect the acquisition of specific immunity in pediatric patients of a certain age.

Furthermore, the inclusion of additional control groups, such as SARS-CoV-2-infected adults or uninfected children and adolescents, would have enhanced the interpretability of our data and contextualized pediatric immune responses more effectively. Although comparisons with age-specific reference ranges for the available immune parameters (i.e., major lymphocyte subsets or proliferative capacity), offer a useful benchmark, the absence of a contemporaneous, the absence of age-matched control population limits the strength of causal inferences and between-group comparisons in our study. Of note, though, the inclusion of healthy pediatric controls is inherently constrained by ethical considerations, particularly the imperative to avoid non-essential testing in children, and by the general hesitancy of families to participate, which was heightened at the time of the study by the ongoing pandemic situation. Additionally, our recruitment site was a pediatric hospital, considerably limiting access to adult participants for this purpose.

Despite these constraints, we employed a diverse set of immunological assays to provide complementary insights into each patient’s immune profile. This multidimensional approach was intended to mitigate limitations in cohort size and composition and to support the robustness of our findings within the context of available resources.

## 5. Conclusions

Since the beginning of the pandemic, there has been exponential growth in our perception of the interplay between SARS-CoV-2 and the immune system in children and adult populations. Still, the underlying immune pathology of severe conditions and other complications like MIS-C requires consolidation [29,58]. This work supports many others, highlighting that there are important deviations seen not only for antibody production, often used as the most immediate marker for specific responses, but also in cellular subsets and specific cellular responses to the virus. For instance, we described the immune dynamics of infection, which somehow follow the patterns observed in adults, with the notoriety of ASCs and T-cell activation and exhaustion in acute disease, and the development over time of specific humoral and cellular responses.

Furthermore, we underline the lower magnitude of these responses in younger patients, who also showed important activation signals, though without the same levels of exhaustion seen in adolescents. Finally, differences could also be encountered in children with severe complications, usually younger, and predominantly boys, showing altered T/B-cell ratios, and reduced specific and unspecific responses at infection.

We hope that the identification of age-, gender-, and disease severity-dependent variations can be helpful for patient management, as well as the identification of biomarkers of disease severity and associated complications. Moreover, a better comprehension of immune mechanisms in young patients can also be useful when designing immunization strategies adjusted to different age groups.

## Figures and Tables

**Table 1:** Recommendations from the Directorate-General for Health used when approaching patients with COVID-19 in the present study.

**Asymptomatic Infection**	
**Mild disease**	Mild symptoms, including anosmia, ageusia, or dysgeusia of sudden onset
**Moderate disease**	Fever lasting 3 or more days; or, Dyspnea ^1^ but with SpO_2_ ≥ 90% (or ≥ 93% in pediatric age) on room air, and without hemodynamic instability
**Severe disease**	Pneumonia ^2^ with respiratory distress, respiratory rate > 30 cpm, or SpO_2_ < 90% on air room; or with hemodynamic instability

Critical disease is considered in the presence of ARDS, sepsis, septic shock, major vascular events, or pulmonary thromboembolism, MIS-C, myocarditis, or encephalitis. ^1^ In children, assess respiratory distress, subcostal, suprasternal, and intercostal indrawing, nasal flaring, and tachypnea. ^2^ In children, assess tachypnea, hypoxemia, groaning, rib indrawing, cyanosis, feeding difficulty, dehydration, prostration, altered consciousness, seizures, or involuntary movements.

**Table 2:** Demographic and clinical characteristics of children and adolescents enrolled in the study.

Group	T1 *n* = 74	T2 *n* = 23	T3 *n* = 20	T4 *n* = 28	*p*-Value
**Gender, n (%)**					0.0196 ^a^
Girls	35 (47.3)	8 (34.7)	2 (10.0)	13 (46.4)	
Boys	39 (52.7)	15 (65.2)	18 (90.0)	15 (53.6)	
	^#^ vs. ^T3^		^#^ vs. ^T4^		
**Age, y**					0.5293 ^b^
Median	6.3	8.4	2.8	6.7	
[IQR]	[1.3–13.6]	[3.9–13.3]	[0.7–12.5]	[1.7–10.6]	
[Range]	[0.0–17.8] *	[0.3–17.9]	[0.4–17.6]	[0.7–17.8]	
**Time post-infection**					<0.0001 ^b^
**Months,**	0.1 (3.0 days)	2.2	4.4	8.6	
Median [IQR]	[0.0–0.2]	[1.2–2.5]	[3.5–5.3]	[7.1–10.6]	
**Disease symptoms, n (%)**	(*n* = 73)				0.5682 ^a^
Asymptomatic	25 (34.2)	7 (30.4)	4 (20.0)	8 (28.6)	
Mild	23 (31.5)	7 (30.4)	5 (25.0)	6 (21.4)	
Moderate	7 (9.5)	0 (0.0)	2 (10.0)	2 (7.1)	
Severe	18 (24.3)	9 (39.1)	9 (45.0)	12 (42.9)	

Legend: IQR, interquartile range; KW, Kruskal–Wallis; mo, months; y, years. ^a^ Chi-square test. ^b^ Kruskal–Wallis test. T1: time point 1, (<14 d of infection); T2: time point 2, (14 d to 3 months after infection); T3: time point 3, (3 to 6 months after infection); T4: time point 4, (6 to 12 months after infection). * Age range in T1 goes from 7 days to 17 years. ^#^ *p*-value < 0.05, Fisher’s exact test.

**Table 3:** Demographic characteristics of children and adolescents enrolled at T1, grouped according to symptom severity.

**Symptoms at T1**	**Asymptomatic** ***n* = 25**	**Mild** ***n* = 23**	**Mod-sev** ***n* = 21**	**MIS-C** ***n* = 4**	*p*-Value *
**Gender, n (%)**					0.0250 ^a^
**Girls**	17 (68.0)	8 (34.8)	7 (34.6)	2 (50.0)	
**Boys**	8 (32.0)	15 (65.2)	14 (65.4)	2 (50.0)	
	^#^ vs. ^Mild^ ^#^ vs. ^Mod-Sev^	^n.s.^ vs. ^Mod-sev^			
**Age, years** **Median [IQR]**	11.9 [5.5–15.7] ^*p* = 0.0020^ vs. ^Mild^	1.7 [0.2–10.1]	5.9 [1.3–12.9]	1.4 [0.5–8.4]	0.0023 ^b^

Legend: ^a^ Chi-square; ^b^ ANOVA, followed by Holm–Šídák’s multiple comparisons test; MIS-C, Multisystem Inflammatory Syndrome in Children; mod, moderate; sev, severe; T1, time point 1. n.s., non significant, Fisher’s exact test; ^#^, *p*-value < 0.05, Fisher’s exact test. * The MIS-C group was excluded from *p*-value calculations.

## Data Availability

Data supporting the findings of this study are available from the corresponding authors upon reasonable request.

## Supplementary Materials

The following supporting information can be downloaded at: https://www.mdpi.com/article/10.3390/pathogens14090838/s1, Figure S1. Inclusion and exclusion criteria for the enrollment process; Figure S2. SARS-CoV-2 specific responses along the 4 time points of the study, according to patient age, gender, and symptom severity; Table S1. Patient characteristics at infection, and subsequential timepoints; Table S2. Immune profile characterized in patients along the 4 time points of the study; Table S3. Complete immune profile characterized in patients from all 4 time points of the study.

## List of Abbreviations

ACE-2	Angiotensin-Converting Enzyme 2
ASC	antibody-secreting cells
CHULC	Centro Hospitalar Universitário de Lisboa Central
COVID-19	Coronavirus Disease 2019
EDTA	Ethylenediaminetetraacetic acid
IFN	interferon
MFI	mean fluorescence intensity
MIS-C	Multisystem Inflammatory Syndrome in Children
NK	Natural killer cells
NS	non-stimulated
PCR	Polymerase Chain Reaction
PD-1	Programmed cell Death protein 1
PHA	Phytohemagglutinin
SARS-CoV-2	Severe Acute Respiratory Syndrome Coronavirus 2
SI	stimulation index
T1	time point 1, (<14 d of infection)
T2	time point 2, (14 d to 3 months after infection)
T3	time point 3, (3 to 6 months after infection)
T4	time point 4, (6 to 12 months after infection)
Tch	follicular CD8+ T cytotoxic cells
Tfh	follicular CD4+ T helper cells
Tr	transitional
UNICEF	United Nations International Children’s Emergency Fund
WHO	World Health Organization
