# Supplementary material for: SARS-CoV-2 Infection in Children: Revisiting Host–Virus Interactions Through Post-Infection Immune Profiling
Source: Pathogens. 2025 Aug 22;14(9):838. doi: 10.3390/pathogens14090838 (PMC12472562; doi:10.3390/pathogens14090838)
Supplement: Supplementary file 1 [file pathogens-14-00838-s001.zip › pathogens-3783567_Supplementary Figure 1_v.01.pdf]

Supplementary Figure S1: Inclusion and Exclusion criteria for the enrollment process

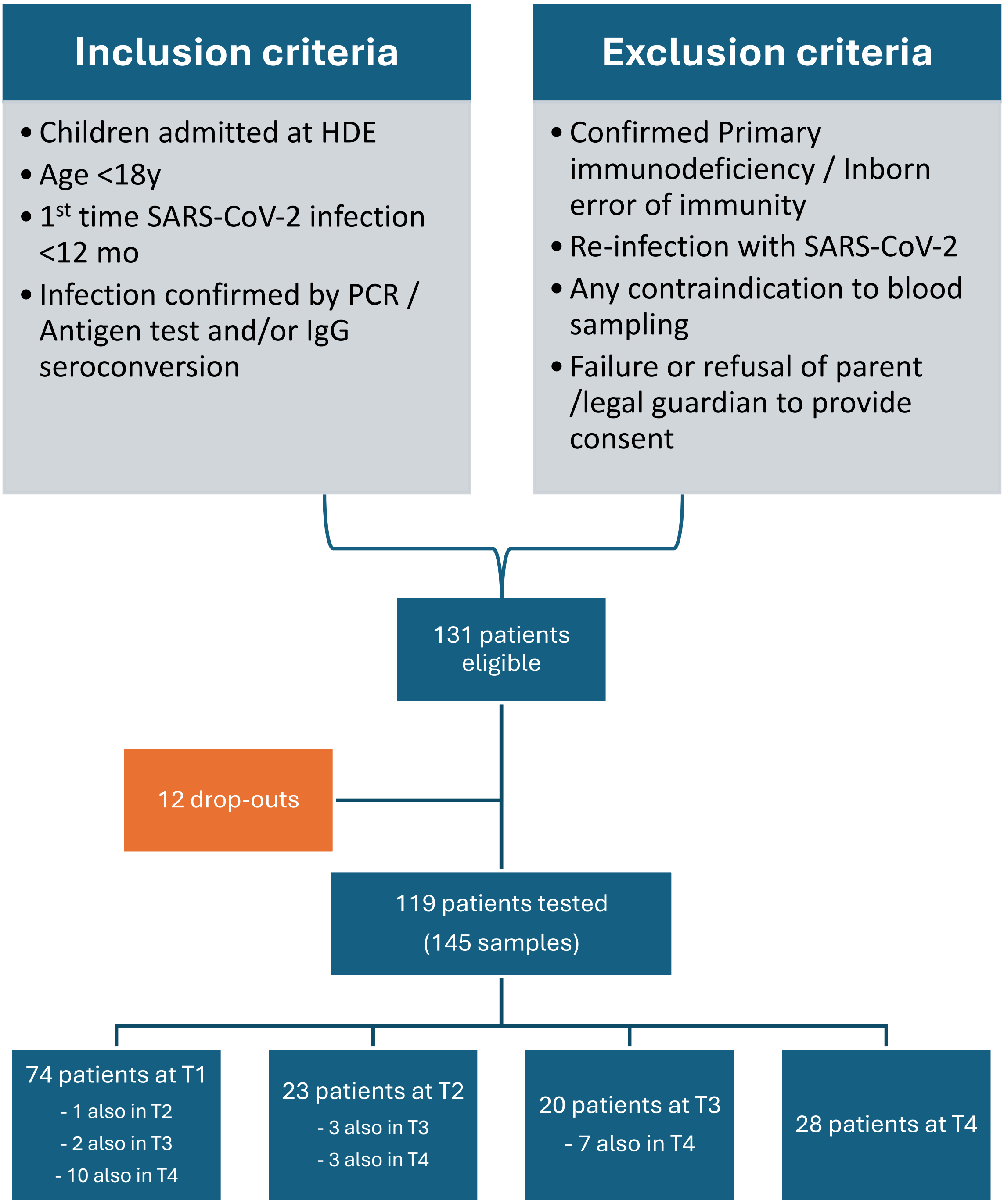

Legend: HDE – Hospital D. Estefânia; mo, months; PCR - Polymerase Chain Reaction; SARS-CoV-2, Severe Acute Respiratory Syndrome Coronavirus; y, years.
