# Supplementary material for: SARS-CoV-2 Infection in Children: Revisiting Host–Virus Interactions Through Post-Infection Immune Profiling
Source: Pathogens. 2025 Aug 22;14(9):838. doi: 10.3390/pathogens14090838 (PMC12472562; doi:10.3390/pathogens14090838)
Supplement: Supplementary file 1 [file pathogens-14-00838-s001.zip › pathogens-3783567_Supplementary Figure 2_v.01.pdf]

Supplementary Figure S2: SARS-CoV-2 specific responses along the 4 time points of the study, according to patient age, gender, and symptom severity

a. Gender

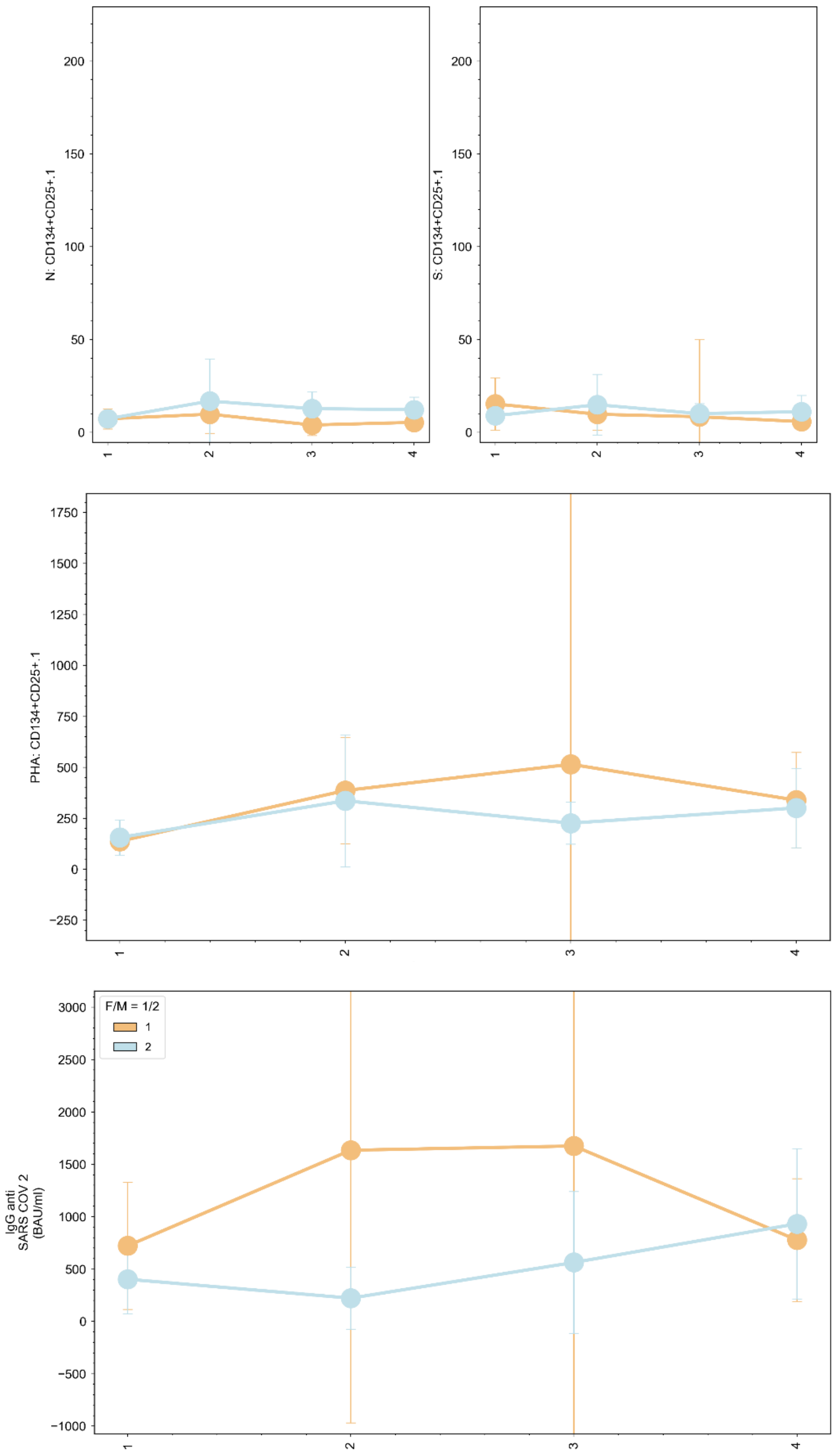

Legend: F, female (rose); M, male (light blue); 1, Time point 1; 2, Time point 2; 3, Time point 3; 4, Time point 4.

b. Age Group

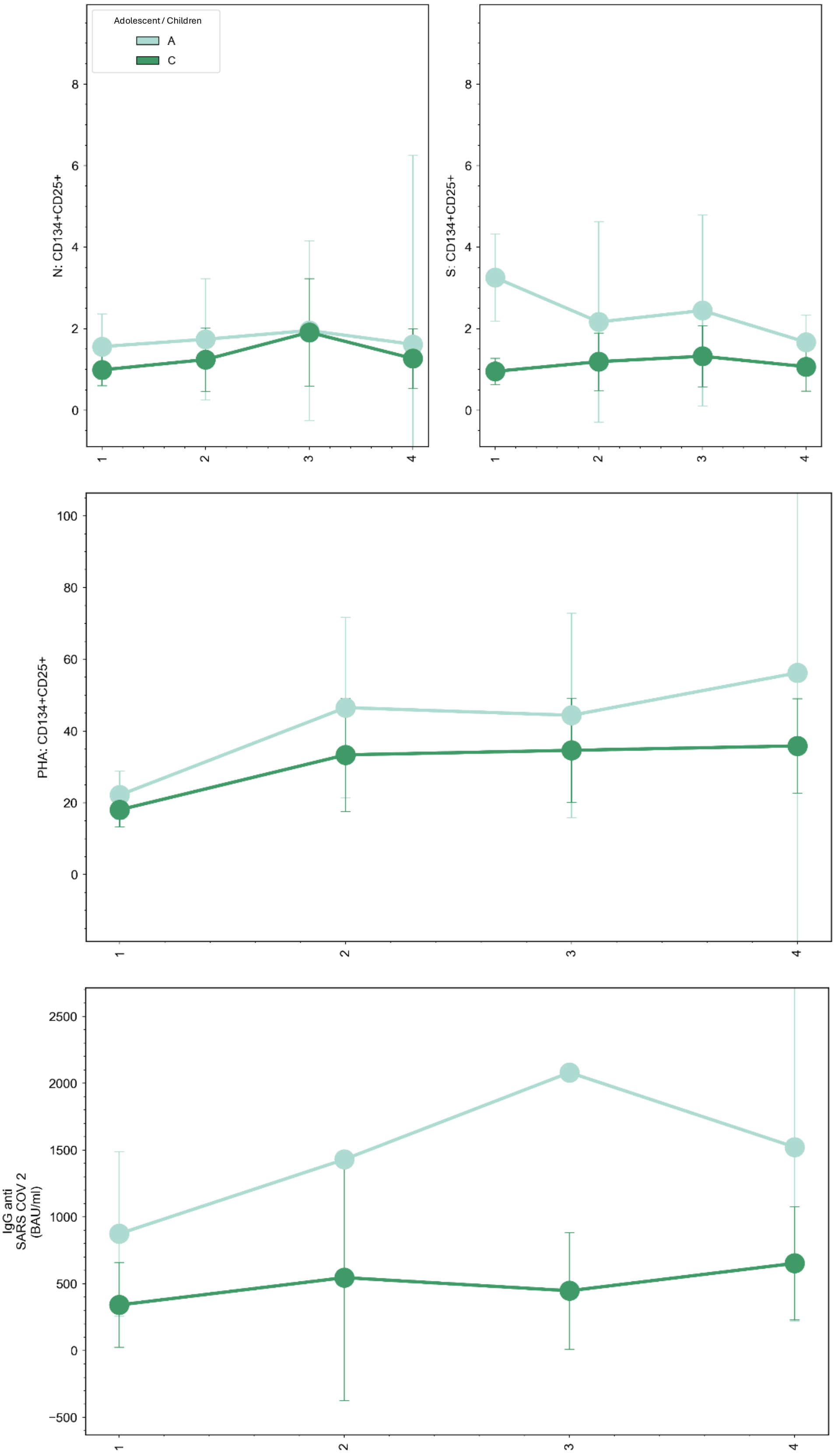

Legend: A, Adolescents (light green); C, Children (green); 1, Time point 1; 2, Time point 2; 3, Time point 3; 4, Time point 4.

c. Severity of Symptoms

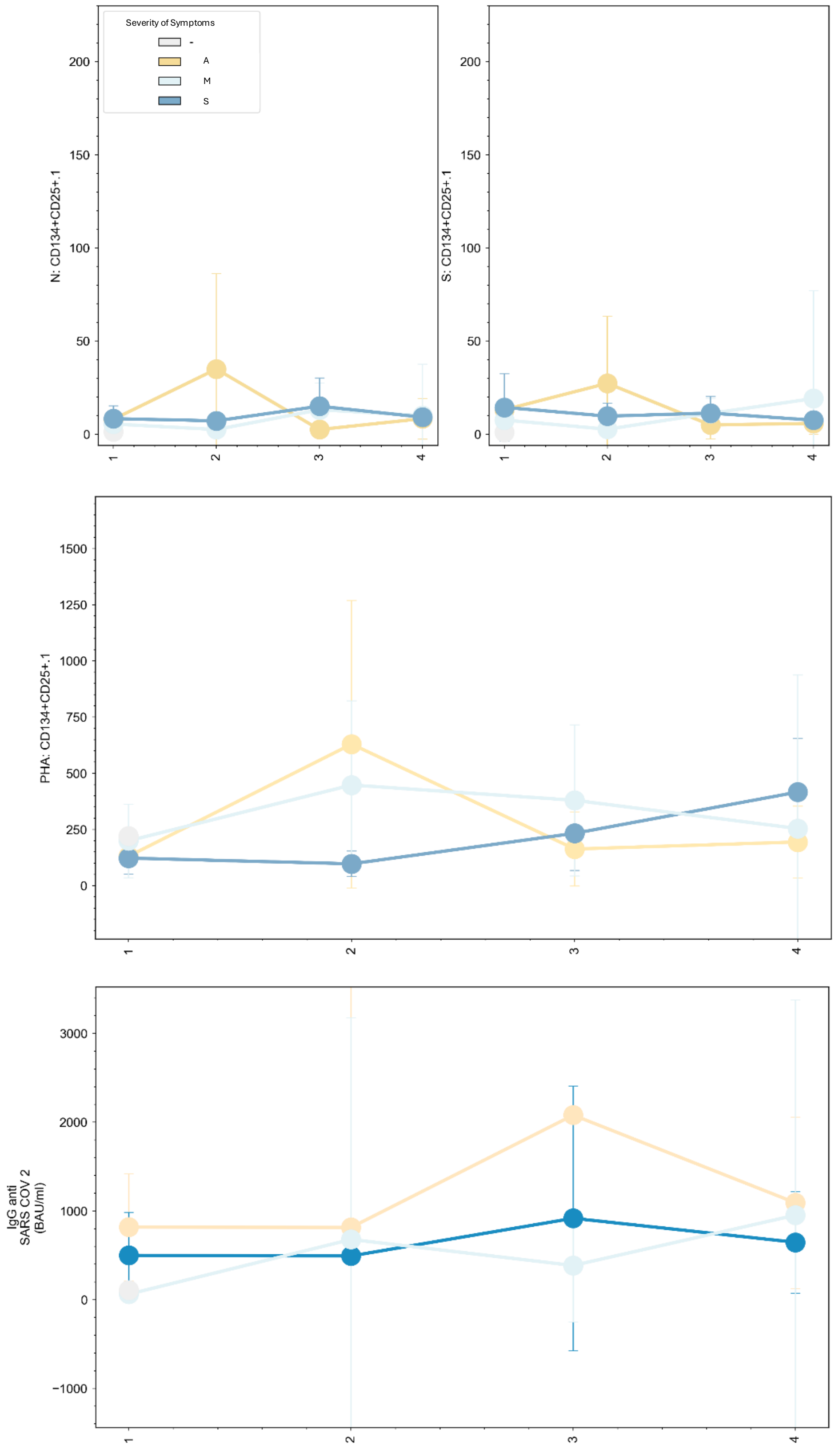

Legend: A - Asymptomatic (rose); M - Mild (light blue); S - Severe (dark blue); 1, Time point 1; 2, Time point 2; 3, Time point 3; 4, Time point 4.
