# Supplementary material for: SARS-CoV-2 Infection in Children: Revisiting Host–Virus Interactions Through Post-Infection Immune Profiling
Source: Pathogens. 2025 Aug 22;14(9):838. doi: 10.3390/pathogens14090838 (PMC12472562; doi:10.3390/pathogens14090838)
Supplement: Supplementary file 1 [file pathogens-14-00838-s001.zip › pathogens-3783567_Supplementary Table 1_v.01.pdf]

Supplementary Table S1 - Patient characteristics at Infection, and subsequential timepoints

Patient (T1)	Gender	Age (Years)	Time after infection (days)	Time after infection (months)	Cause of Hospital Admission	Symptom severity	Posterior Development of MIS-C
1	M	0,02	4,00	0,13	Leukopenia, neutropenia, thrombocytopenia	Mild	No
2	F	0,04	4,00	0,13	Moaning + feeding refusal	Mild	No
3	M	0,08	1,00	0,03	Nasal obstruction + cough + feeding difficulty in newborn	Mild	No
4	F	0,08	2,00	0,07	Fever + intermittent moaning + nasal obstruction + decreased activity + diarrhea	Mild	No
5	M	0,15	0,00	0,00	Fever + ARDS + hypoxemia	Severe	No
6	M	0,17	4,00	0,13	Pneumococcal meningitis with COVID-19 during hospitalization	Mild	No
7	F	0,17	4,00	0,13	Cough + feeding refusal	Mild	No
8	M	0,18	6,00	0,20	Cough + rhinorrhea + fever + irritability --> viral sepsis	Severe	No
9	M	0,20	4,00	0,13	Fever + ARDS + hypoxemia	Severe	No
10	F	0,32	0,00	0,00	COVID-19 pneumonia	Severe	Yes
11	F	0,41	2,00	0,07	Fever + hepatitis	Mild	No
12	M	0,68	2,00	0,07	Cough and rhinorrhea	Mild	No
13	M	0,88	9,00	0,30	Fever + cough + rhinorrhea	Mild	No
14	M	0,92	2,00	0,07	COVID-19 pneumonia with hypoxemia	Severe	No
15	F	0,96	5,00	0,17	Fever + feeding refusal + dehydration + febrile seizure	Mild	No
16	F	1,12	6,00	0,20	COVID-19 pneumonia	Severe	Yes
17	M	1,14	1,00	0,03	Cough + rhinorrhea	Mild	No
18	M	1,28	3,00	0,10	ARDS + fever + hypoxemia	Severe	No
19	F	1,31	8,00	0,27	ARDS + fever + hypoxemia	Severe	No
20	F	1,49	2,00	0,07	Burn	Asymptomatic	No
21	M	1,60	7,00	0,23	COVID-19 pneumonia	Severe	Yes
22	F	1,62	2,00	0,07	Rotine follow up	Asymptomatic	No
23	M	1,68	1,00	0,03	Fever + cough + feeding refusal	Mild	No
24	M	1,68	14,00	0,47	Nosocomial infection	Asymptomatic	No
25	F	2,24	4,00	0,13	COVID-19 pneumonia	Moderate	No
26	M	2,61	2,00	0,07	Fever + rhinorrhea + cough	Mild	No
27	M	3,00	3,00	0,10	Rhinorrhea + nasal obstruction + fever	Mild	No
28	M	3,13	3,00	0,10	Status epilepticus	Mild	No
29	F	3,26	6,00	0,20	Fever + cough + rhinorrhea + hypoxemia	Severe	No
30	F	3,28	1,00	0,03	Burn	Asymptomatic	No
31	M	3,69	1,00	0,03	Fever + ARDS + hypoxemia	Moderate	No
32	M	3,79	5,00	0,17	Mandibular fracture	Asymptomatic	No
33	M	4,99	4,00	0,13	COVID-19 pneumonia	Severe	No
34	F	5,24	8,00	0,27	Nosocomial infection	Asymptomatic	No
35	M	5,68	2,00	0,07	Rotine follow up	Asymptomatic	No
36	F	5,86	7,00	0,23	Fever + hepatitis + ARDS	Moderate	No
37	M	6,00	3,00	0,10	Fever + cough + rhinorrhea	Mild	No
38	M	6,53	4,00	0,13	Fever + cough	Mild	No
39	M	6,77	7,00	0,23	Fulminant acute cerebellitis	Severe	No
40	F	7,04	3,00	0,10	Arthritis	Artrite pos COVID19	No
41	F	8,17	10,00	0,33	Altered level of consciousness	Severe	No
42	F	8,25	1,00	0,03	Arthritis	N/D	No
43	M	9,11	4,00	0,13	Dental abscess	Asymptomatic	No
44	M	10,04	1,00	0,03	ARDS + hypoxemia + cough + fever	Severe	No
45	F	10,06	8,00	0,27	Appendicitis	Asymptomatic	No
46	F	10,07	3,00	0,10	Headaches, fainting, myalgia, and persistent vomiting	Mild	No
47	M	10,09	2,00	0,07	Acute gastroenteritis due to Campylobacter	Asymptomatic	No
48	F	10,58	2,00	0,07	Lobar pneumonia + acute thoracic syndrome	Asymptomatic	No
49	M	10,73	1,00	0,03	COVID-19 pneumonia	Severe	Yes
50	F	11,33	8,00	0,27	Rotine follow up	Asymptomatic	No
51	F	11,86	5,00	0,17	Acute appendicitis	Asymptomatic	No
52	F	12,04	1,00	0,03	Fever + cough + nasal obstruction	Mild	No
53	M	12,08	2,00	0,07	COVID-19 pneumonia	Moderate	No
54	M	12,58	5,00	0,17	Fever + mesenteric adenitis + enteritis	Mild	No
55	F	13,21	2,00	0,07	Rotine follow up	Asymptomatic	No
56	F	13,56	2,00	0,07	Rotine follow up	Asymptomatic	No
57	M	13,67	5,00	0,17	COVID-19 pneumonia	Moderate	No
58	M	14,09	1,00	0,03	Acute appendicitis	Asymptomatic	No
59	M	14,29	1,00	0,03	Nosocomial infection	Mild	No
60	F	14,34	3,00	0,10	Nosocomial infection	Asymptomatic	No
61	M	14,65	0,00	0,00	Testicular torsion	Asymptomatic	No
62	F	15,25	2,00	0,07	Chronic kidney disease	Asymptomatic	No
63	M	16,07	1,00	0,03	Gunshot wound	Asymptomatic	No
64	F	16,18	2,00	0,07	Rotine follow up	Asymptomatic	No
65	F	16,26	1,00	0,03	Elective admission for IV therapy	Asymptomatic	No
66	M	16,34	1,00	0,03	Spontaneous pneumothorax	Mild	No
67	F	16,35	5,00	0,17	Urinary tract infection + Chronic kidney disease worsening	Asymptomatic	No
68	F	16,48	3,00	0,10	Polytraumatized	Asymptomatic	No
69	F	16,96	2,00	0,07	Rotine follow up	Asymptomatic	No
70	M	16,96	2,00	0,07	Signs of respiratory distress + acute thoracic syndrome	Moderate	No
71	M	17,20	0,00	0,00	ARDS + fever + hypoxemia	Severe	No
72	M	17,55	8,00	0,27	Fever + rhinorrhea + cough	Moderate	No
73	F	17,69	5,00	0,17	ARDS + fever + hypoxemia	Severe	No
74	F	17,76	1,00	0,03	Mild cough – peritonsillar abscess	Mild	No

Patient (T2)	Gender	Age (Years)	Time after infection (days)	Time after infection (months)	Cause of Hospital Admission	Symptom severity	Posterior Development of MIS-C
1	M	0,31	N/A	2,47	Fever + feeding refusal + bicytopenia	Mild	
2	M	0,75	N/A	2,47	Asymptomatic – Acute appendicitis	Asymptomatic	
3	F	0,95	N/A	2,47	Adenophlegmon	Mild	
4	M	1,89	N/A	1,27	Fever + cough + rhinorrhea	Mild	
5	M	2,45	N/A	1,70	MIS-C	Severe	Yes
6	M	3,85	N/A	2,47	Cough + rhinorrhea	Mild	
7	M	4,15	N/A	2,23	Cardiopathy with heart failure	Mild	
8	M	5,22	N/A	0,80	Fever with nodular eschar	Asymptomatic	
9	M	5,57	N/A	3,00	MIS-C	Severe	Yes
10	M	7,23	N/A	1,27	MIS-C	Severe	Yes
11	M	7,81	N/A	1,13	MIS-C	Severe	Yes
12	F	8,44	N/A	3,00	MIS-C	Severe	Yes
13	F	9,68	N/A	2,63	Asymptomatic – Nosocomial infection	Asymptomatic	
14	M	10,22	N/A	3,00	MIS-C	Severe	Yes
15	M	10,27	N/A	2,23	Asymptomatic – Campylobacter gastroenteritis	Asymptomatic	
16	F	12,27	N/A	2,80	Asymptomatic – Appendicitis	Asymptomatic	
17	M	12,46	N/A	1,77	MIS-C	Severe	Yes
18	M	13,34	N/A	0,80	COVID-19 pneumonia follow up	Severe	
19	M	13,53	N/A	2,53	Cough, respiratory distress, COVID-19 pneumonia with hypoxemia - follow up	Severe	
20	F	14,40	N/A	2,97	Asymptomatic – Appendicitis	Asymptomatic	
21	F	16,34	N/A	0,73	Unconfirmed suspicion of MIS-C – Adenovirus infection	Asymptomatic	
22	F	17,19	N/A	2,27	Acute tonsillitis and bicytopenia	Mild	
23	F	17,91	N/A	1,13	Rhinorrhea, nasal obstruction, cough, odynophagia, headache	Mild	

Patient (T3)	Gender	Age (Years)	Time after infection (days)	Time after infection (months)	Cause of Hospital Admission	Symptom severity	Posterior Development of MIS-C
1	M	0,37	N/A	3,70	Fever in Newborn	Mild	
2	M	0,46	N/A	3,47	Asymptomatic – UTI	Asymptomatic	
3	M	0,51	N/A	5,83	COVID-19 pneumonia - follow up	Moderate	
4	M	0,52	N/A	5,83	Fever + lethargy + grunting – viral sepsis	Severe	
5	M	0,53	N/A	5,10	2nd collection	Mild	
6	M	0,99	N/A	3,30	Asymptomatic – Appendicitis	Asymptomatic	
7	F	1,07	N/A	3,47	MIS-C	Severe	Yes
8	M	1,65	N/A	4,70	Fever + cough + snoring-like sounds + feeding refusal	Mild	
9	M	2,56	N/A	5,80	COVID-19 pneumonia - follow up	Severe	
10	M	2,62	N/A	5,27	COVID-19 with bacterial superinfection – lobar pneumonia - follow up	Mild	
11	M	3,04	N/A	5,27	2nd collection	Mild	
12	M	3,23	N/A	4,60	COVID-19 pneumonia - follow up	Moderate	
13	M	8,07	N/A	4,23	MIS-C	Severe	Yes
14	M	8,64	N/A	4,00	Pneumonia with hypoxemia	Severe	
15	M	11,92	N/A	4,40	MIS-C	Severe	Yes
16	M	12,69	N/A	4,57	MIS-C	Severe	Yes
17	M	13,34	N/A	4,00	MIS-C	Severe	Yes
18	M	16,48	N/A	3,47	COVID-19 pneumonia with hypoxemia - follow up	Severe	
19	F	16,61	N/A	4,00	2nd collection	Asymptomatic	
20	M	17,56	N/A	3,47	Asymptomatic	Asymptomatic	

Patient (T4)	Gender	Age (Years)	Time after infection (days)	Time after infection (months)	Cause of Hospital Admission	Symptom severity	Posterior Development of MIS-C
1	M	0,73	N/A	7,00	2nd collection	Severe	
2	F	0,99	N/A	7,83	MIS-C	MIS-C	Yes
3	M	1,02	N/A	10,27	2nd collection	Asymptomatic	
4	M	1,14	N/A	12,00	2nd collection	Mild	
5	M	1,55	N/A	10,07	2nd collection	Asymptomatic	
6	F	1,62	N/A	10,20	2nd collection	Severe	Yes
7	F	1,74	N/A	12,00	2nd collection	Mild	
8	M	1,74	N/A	12,00	Asymptomatic - urosepsis	Asymptomatic	
9	F	1,87	N/A	7,07	2nd collection	Severe	
10	F	2,19	N/A	7,10	2nd collection	Asymptomatic	
11	M	2,20	N/A	6,37	2nd collection	Mild	
12	M	3,40	N/A	6,70	2nd collection	Moderado	
13	F	4,24	N/A	11,57	MIS-C	Severe	Yes
14	F	6,45	N/A	7,43	2nd collection	Moderate	
15	F	6,89	N/A	10,73	Asymptomatic – Varicella with superinfection	Asymptomatic	
16	M	8,46	N/A	10,50	MIS-C	Severe	Yes
17	F	8,76	N/A	7,53	2nd collection	Severe	
18	M	8,76	N/A	7,00	MIS-C	Severe	Yes
19	M	8,87	N/A	6,80	2nd collection	Severe	
20	F	8,94	N/A	9,00	2nd collection	Severe	Yes
21	M	10,30	N/A	8,57	MIS-C	Severe	Yes
22	F	10,75	N/A	8,67	2nd collection	Asymptomatic	
23	M	13,08	N/A	6,27	2nd collection	Mild	
24	M	13,65	N/A	7,40	Asymptomatic - fracture	Asymptomatic	
25	F	16,98	N/A	8,43	2nd collection	Asymptomatic	
26	M	17,13	N/A	9,63	2nd collection	Mild	
27	M	17,13	N/A	11,43	2nd collection	Severe	
28	F	17,79	N/A	9,50	2nd collection	Mild	
