# Supplementary material for: SARS-CoV-2 Infection in Children: Revisiting Host–Virus Interactions Through Post-Infection Immune Profiling
Source: Pathogens. 2025 Aug 22;14(9):838. doi: 10.3390/pathogens14090838 (PMC12472562; doi:10.3390/pathogens14090838)
Supplement: Supplementary file 1 [file pathogens-14-00838-s001.zip › pathogens-3783567_Supplementary Table 2_v.01.pdf]

Supplementary Table S2

Panels of Monoclonal Antibodies used in the different immunophenotyping and functional assays performed in the study

Tube 1 – EXBIO DryFlowEx ACT T Screen kit<br>Cat. No. ED7705							
CD4<br>FITC	PD-1/CD279<br>PE	HLA-DR<br>PerCP™ Cy5.5	CD3<br>PE™ Cy7	CXCR5/CD185<br>APC	CD38<br>APC-Cy™ 7	CD45<br>Pacific Blue™	CD8<br>Pacific Orange™
Clone MEM-241	Clone EH12.2H7	Clone L243	Clone SK7	Clone J252D4	Clone HIT2	Clone 2D1	Clone MEM-31
Tube 2 – DryFlowEx ASC Screen kit<br>Cat. No. ED7704							
IgD<br>FITC	CD27<br>PE	CD24<br>PerCP™ Cy5.5	CD19<br>PE™ Cy7	CD21<br>APC	CD38<br>APC-Cy™ 7	CD45<br>Pacific Blue™	-
Clone IA6-2	Clone LT27	Clone SN3	Clone LT19	Clone LT21	Clone HIT2	Clone 2D1	-
Tube 3 – Cytognos ACT-T4 Cell™ kit<br>Cat. No. CYT-AT4C							
CD4<br>FITC	CD134<br>PE	CD3<br>PerCP™ Cy5.5	-	CD25<br>APC	-	-	-
Clone RPA-T4	Clone 134-1	Clone UCHT-1	-	Clone M-A251	-	-	-
