# Supplementary material for: SARS-CoV-2 Infection in Children: Revisiting Host–Virus Interactions Through Post-Infection Immune Profiling
Source: Pathogens. 2025 Aug 22;14(9):838. doi: 10.3390/pathogens14090838 (PMC12472562; doi:10.3390/pathogens14090838)
Supplement: Supplementary file 1 [file pathogens-14-00838-s001.zip › pathogens-3783567_Supplementary Table 3.pdf]

Supplementary Table S3
Complete immune profile characterized in patients from all 4 time points of the study

Group	T1 (n=74)<br>Active infection, <14d	T2 (n=23)<br>14d-3mo after infection	T3 (n=20)<br>3-6mo after infection	T4 (n=28)<br>> 6mo after infection	p-value
**T cell Compartment**					
T cells (% of Lymphocytes)	69,5 [63,4; 76,9]	70,7 [64,9; 75,1]	63,6 [59,1; 75,0]	67,5 [64,4; 77,0]	n.s. *
CD4 T cells (% of T cells)	60,1 [53,3; 66,7]	53,3 [42,1; 60,2]	56,7 [46,3; 70,9]	53,5 [51,4; 59,3]	n.s. *
Activated CD4 T cells (% of CD4):<br>- CD38+ HLADR+	2,4 [1,6; 3,7]	2,2 [1,6; 2,7]	2,4 [1,7; 3,9]	2,0 [1,5; 3,1]	n.s. *
- PD-1 expression (MFI)	683,5 [512,0; 933,8]	562,0 [479,0; 744,0]	557,0 [446,0; 715,0]	630,0 [486,0; 764,0]	n.s. *
- CD38+ HLA-DR-	71,1 [57,4; 86,3]	65,3 [52,0; 78,6]	80,4 [52,5; 91,1]	79,3 [67,8; 85,0]	n.s. *
- CD38- HLA-DR+	1,9 [0,6; 2,8]	2,1 [1,4; 2,9]	1,5 [0,8; 5,8]	1,7 [0,9; 2,5]	n.s. *
- PD-1 expression MFI	507,5 [418,8; 605,5]	455,0 [427,0; 553,0]	457,0 [401,0; 497,0]	439,0 [374,0; 486,0]	0,0183 *
- CD38- HLA-DR-	23,7 [10,8; 35,6]	28,9 [17,0; 41,9]	16,2 [6,2; 38,5]	16,1 [9,5; 29,8]	n.s. *
Follicular CD4 T cells (% of CD4):<br>- CXCR5+ PD-1+	5,6 [3,6; 7,3]	6,1 [4,3; 7,7]	3,8 [2,6; 6,1]	5,6 [4,1; 6,4]	n.s. *
- CD38 expression MFI	1373,0 [1000,0; 2378,0]	1043,0 [792,0; 1430,0]	1529,0 [886,0; 2088,0]	1361,0 [870,0; 2132,0]	n.s. *
- HLA DR expression MFI	353,0 [282,5; 488,0]	351,0 [303,0; 486,0]	457,0 [406,0; 537,0]	315,0 [263,0; 419,0]	0,0094 *
- CXCR5+ PD-1-	5,4 [3,7; 7,6]	7,8 [3,9; 10,7]	4,6 [1,8; 8,3]	6,1 [3,3; 8,3]	n.s. *
- CXCR5- PD-1+	15,3 [8,2; 22,5]	12,3 [9,0; 20,1]	8,8 [6,1; 24,2]	13,0 [7,5; 14,8]	n.s. *
CD8 T cells	32,1 [25,5; 37,6]	33,1 [27,2; 39,8]	31,7 [24,5; 39,5]	33,2 [29,1; 36,8]	n.s. *
Activated CD8 T cells (% of CD8)<br>- CD38hi/+ HLA-DR+	9,8 [4,5; 17,7]	4,7 [3,1; 9,8]	6,2 [2,7; 13,5]	5,3 [3,0; 11,8]	n.s. *
- CD38hi/+ HLA-DR-	54,0 [37,1; 67,1]	44,2 [35,0; 55,0]	47,5 [29,6; 79,4]	57,3 [38,8; 68,1]	n.s. *
CD38hi HLA-DR+	2,8 [1,4 ; 5,8]	0,9 [0,5 ; 2,3]	1,2 [0,6 ; 5,2]	0,8 [0,3 ; 5,3]	0,0009 *
- PD-1 expression (MFI)	745,5 [571,0; 1012,0]	679,0 [526,0; 930,0]	549,0 [444,0; 926,0]	716,0 [589,0; 950,0]	n.s. *
CD38+ HLA-DR+	6,1 [2,7; 9,3]	3,1 [2,2; 7,4]	4,6 [2,0; 11,9]	3,9 [2,5; 7,8]	n.s. *
- PD-1 expression (MFI)	410,0 [306,5; 614,8]	470,0 [367,0; 586,0]	358,0 [190,0; 469,0]	415,0 [317,0; 564,0]	n.s. *
- CD38- HLA-DR+	1,3 [0,4; 2,8]	2,8 [1,0; 5,0]	1,4 [0,7; 8,0]	2,1 [1,4; 2,7]	0,0335 *
- PD-1 expression (MFI)	338,0 [237,8; 527,8]	405,0 [366,0; 473,0]	355,0 [222,0; 569,0]	345,0 [293,0; 505,0]	n.s. *
Follicular CD8 T cells (% of CD8)<br>- CXCR5+ PD-1+	1,6 [0,9; 2,2]	1,5 [0,8; 2,2]	1,4 [0,7; 2,2]	1,6 [1,0; 2,2]	n.s. *
- CXCR5+ PD-1-	1,0 [0,7; 1,3]	1,0 [0,7; 1,4]	1,3 [0,9; 1,4]	1,0 [0,8; 1,3]	n.s. *
- CXCR5- PD-1+	23,4 [14,0; 34,0]	21,5 [17,3; 30,5]	20,9 [11,6; 26,1]	22,8 [18,3; 34,3]	n.s. *
T DN	6,9 [4,7; 10,2]	11,8 [6,5; 14,9]	9,4 [7,1; 15,6]	10,0 [7,6; 16,9]	0,0008 *

**B cell compartment**					
B lymphocytes (% of Lymphocytes)	18,6 [13,1; 27,3]	18,0 [13,1; 22,8]	20,8 [13,9; 26,6]	16,8 [13,5; 20,1]	n.s. *
Pre-Germinal center (GC) (% of B)	81,2 [70,5; 87,9]	75,8 [66,4; 82,9]	83,3 [75,9; 90,1]	72,9 [65,6; 80,4]	0.0397 *
Pre-GC/Transitional Tr1-Tr2 (% of B)	11,6 [5,0; 19,2]	7,0 [4,6; 11,8]	13,3 [7,2; 19,3]	9,9 [8,2; 16,0]	n.s. *
Pre-GC/Naive-Tr3 (% of B)	88,4 [80,9; 95,0]	93,0 [88,2; 95,4]	86,7 [80,7; 92,8]	90,1 [84,0; 91,8]	n.s. *
Post-Germinal Center (GC) (% of B)	16,8 [9,9; 26,2]	21,0 [13,4; 29,9]	13,8 [9,1; 19,2]	25,3 [16,1; 27,6]	0.0362 *
Unswitched Memory (% of B)	5,3 [3,8; 8,5]	10,2 [6,7; 12,8]	6,3 [3,9; 9,3]	9,0 [7,1;13,5]	0,0002 *
Switched Memory (% of B)	9,7 [5,5; 18,2]	10,7 [5,8; 16,3]	8,6 [2,3; 11,8]	12,0 [8,3; 15,6]	n.s. *
ASC - Post-GC/Total PB (% of B)	28,7 [15,6; 42,2]	9,2 [3,4; 14,6]	14,1 [9,8; 24,3]	11,1 [3,9; 35,2]	<0,0001*
ASC - Post-GC/early PB (% of PB)	32,6 [22,1; 41,4]	39,0 [24,3; 47,4]	42,8 [25,5; 52,2]	35,7 [32,4; 45,9]	0,0293 *
ASC - Post-GC/mature PB (% of PB)	67,5 [58,6; 77,9]	61,0 [52,6; 75,7]	57,2 [47,8; 74,5]	64,3 [54,1; 67,6]	0,0293 *
Double Negative (DN) (% of B)	2,6 [1,7; 4,1]	3,0 [1,8; 3,8]	1,9 [1,4; 3,7]	3,1 [1,6; 4,1]	n.s. *
**Specific SARS-CoV 2 responses**					
CD25+ CD134+ CD4 T cells					
Response to N protein (%)	0,5 [0,3; 1,2]	1,1 [0,5; 2,2]	0,9 [0,4; 3,8]	0,7 [0,4; 1,6]	n.s. *
Response to S protein (%)	0,9 [0,4; 2,6]	1,3 [0,3; 2,3]	1,2 [0,5; 2,6]	0,8 [0,4; 1,5]	n.s. *
Response to PHA (%) - unspecific	14,1 [7,4; 33,0]	36,5 [12,0; 55,2]	31,5 [21,3; 52,3]	32,6 [14,9; 71,3]	0,0024 *
Response to N protein (SI)	2,8 [1,4; 7,9]	8,0 [3,9; 12,2]	4,4 [2,2; 15,3]	5,1 [2,2; 12,9]	0,0311 *
Response to S protein (SI)	3,9 [1,8; 11,0]	7,4 [3,7; 17,9]	8,2 [3,3; 13,6]	5,3 [2,0; 13,2]	n.s. *
Response to PHA (SI) - unspecific	79,6 [36,2; 200,4]	172,3 [82,5; 477,2]	187,5 [110,0; 315,0]	192,2 [112,4; 435,9]	0,0022 *
SARS-CoV2 IgG (BAU/mL)	62,0 [7,5; 596,8]	239,0 [108,3; 1196,0]	566,0 [113,5; 1675,0]	426,0 [244,8; 1803,0]	0,0505 *

Legend: ASC, antibody secreting cells; GC, Germinal center; *, Kruskal-Wallis test; MFI, Mean Fluorescence Intensity; mo, months; n.s., non-significant; PB, plasmablasts; T, time point; Tr, transitional.
